# Biclustering analysis of transcriptome big data identifies condition-specific microRNA targets

**DOI:** 10.1093/nar/gkz139

**Published:** 2019-03-01

**Authors:** Sora Yoon, Hai C T Nguyen, Woobeen Jo, Jinhwan Kim, Sang-Mun Chi, Jiyoung Park, Seon-Young Kim, Dougu Nam

**Affiliations:** 1 School of Life Sciences, Ulsan National Institute of Science and Technology, Ulsan 44919, Republic of Korea; 2 School of Computer Science and Engineering, Kyungsung University, Busan 48434, Republic of Korea; 3 Department of Functional Genomics, University of Science and Technology (UST), Daejeon 34141, Republic of Korea; 4 Genome Editing Research Center, Personalized Genomic Medicine Research Center, Korea Research Institute of Bioscience and Biotechnology (KRIBB), Daejeon 34141, Republic of Korea; 5 Department of Mathematical Sciences, Ulsan National Institute of Science and Technology, Ulsan 44919, Republic of Korea

## Abstract

We present a novel approach to identify human microRNA (miRNA) regulatory modules (mRNA targets and relevant cell conditions) by biclustering a large collection of mRNA fold-change data for sequence-specific targets. Bicluster targets were assessed using validated messenger RNA (mRNA) targets and exhibited on an average 17.0% (median 19.4%) improved gain in certainty (sensitivity + specificity). The net gain was further increased up to 32.0% (median 33.4%) by incorporating functional networks of targets. We analyzed cancer-specific biclusters and found that the PI3K/Akt signaling pathway is strongly enriched with targets of a few miRNAs in breast cancer and diffuse large B-cell lymphoma. Indeed, five independent prognostic miRNAs were identified, and repression of bicluster targets and pathway activity by miR-29 was experimentally validated. In total, 29 898 biclusters for 459 human miRNAs were collected in the BiMIR database where biclusters are searchable for miRNAs, tissues, diseases, keywords and target genes.

## INTRODUCTION

MicroRNAs (miRNAs) are small non-coding RNA molecules (19–23 nt) that regulate gene expression by binding to miRNA response elements in messenger RNA (mRNA) at the post-transcription level (1,2). Since their discovery, extensive studies have revealed their key roles in regulating cell cycle and differentiation, chronic diseases, cancer progression and other processes (3–6). As the function of an miRNA is characterized by its target genes, there have been efforts to systematically identify these target genes based on the binding sequences (7–12). Although these methods have provided hundreds to thousands of potential targets, they also yield a large number of false-positives and do not suggest specific targets related to the cell condition being examined.

To select more reliable mRNA targets for each miRNA, paired expression profiles of miRNAs and mRNAs (denoted as miRNA–mRNA profiles) have been incorporated considering the anticorrelation between an miRNA and its target mRNA. In addition to simple Pearson and Spearman correlation methods, a number of computational methods that integrate both the binding sequence and miRNA–mRNA profiles have been developed to detect the miRNA–mRNA regulatory relationships including penalized regression and the Bayesian methods (13–15) (denoted as anticorrelation-based methods). Many of these methods used multivariate linear models in which multiple miRNAs regulate a common target gene. Although anticorrelation-based methods have improved target prediction, they require very costly miRNA–mRNA profiles, and only a limited number of such paired datasets are publicly available at present.

Another approach for improving miRNA target prediction is by inference of miRNA regulation modules. Based on binding sequence information, a bipartite graph between miRNAs and mRNAs was constructed and the maximum bicliques (or biclusters) were identified (16,17). These bicliques represent miRNA regulation modules in which multiple miRNAs may coregulate their common targets. By incorporating miRNA–mRNA profiles, these modules were further refined for specific cell conditions (18–21). Because of the modular nature of cellular processes, these modules were considered to represent more reliable miRNA regulation patterns (22). Recent methods incorporated additional information such as protein–protein (or gene–gene) interactions, copy number variation and methylation data to better understand miRNA regulation (23). The myriad of computational methods for miRNA target prediction have been reviewed and categorized previously (15,20,23), some of which are summarized in Supplementary Table S1.

**Figure 1.**
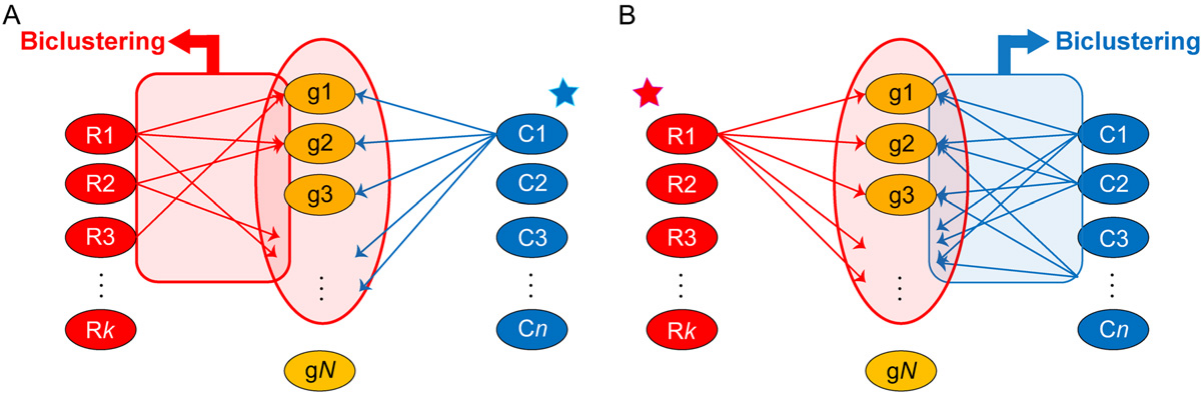
Two approaches for miRNA regulation module discovery. Red, yellow and blue nodes represent miRNA regulators, mRNA target genes and cell conditions, respectively. R, g and C stand for regulator, target gene and cell condition, respectively. (**A**) Existing approach. For a given cell condition (here, C1), down (or up)-regulated mRNAs are selected and biclusters between multiple miRNAs and these mRNA targets are identified. (**B**) Our approach. For a given miRNA (here, R1), mRNAs with corresponding binding sequences are selected and biclusters between these mRNAs and multiple cell conditions are searched.

In this study, we propose a novel approach to identifying miRNA targets for a variety of cell conditions by biclustering a large collection of mRNA profiles for sequence-specific targets. To this end, we collected 5158 human microarray expression datasets with diverse test and control conditions from the Gene Expression Omnibus (GEO) database (24) and compiled corresponding fold-change (FC) profiles representing 5158 cell conditions. Whereas existing methods for miRNA regulation modules biclustered miRNAs and mRNA targets under a given cell condition (Figure 1A), we considered a different dimension and biclustered mRNA targets and cell conditions (i.e. FC profiles) for an miRNA of interest (Figure 1B). Our approach provides more reliable miRNA target groups that are robustly regulated across different cell conditions without using miRNA–mRNA profiles. A related approach incorporated coexpression of sequence-specific targets using 250 microarray datasets to prioritize true targets (25), but it clustered only target genes and did not suggest relevant cell conditions.

Typically, biclustering algorithms seek to identify a complete association (i.e. biclique) between two subsets of nodes (e.g. a subset of target genes and a subset of cell conditions) (26,27). Taking into account the noise in microarray data, we developed a progressive bicluster extension (PBE) algorithm that allows for a small portion of unconnected pairs between two node subsets but yields biclusters of much larger sizes. In the initial step, PBE identifies bicliques using the bimax algorithm (27). These bicliques are used as seeds that are extended by competitively adding ‘dense’ (low proportion of zero values) rows and columns. Next, less dense rows and columns are removed based on a threshold. By increasing this threshold (tight to less tight) during the iteration of bicluster extension, PBE identified the bicluster structures from noisy data more accurately than state-of-the-art algorithms (17,27–31). QUBIC (29) uses a similar approach by searching for seed biclusters that are then extended. However, QUBIC applies a threshold for minimum column density only, which does not change during extension and does not remove noisy rows (Supplementary Figure S4B).

The biclusters were assessed using experimentally validated targets and exhibited substantially improved accuracy compared to the purely sequence-based method. The accuracy was even further improved by selecting the targets having functional interactions with other target genes. Notably, these gains were obtained using only publicly available gene expression and protein functional interaction data, but were compared favorably with those obtained from the anticorrelation-based methods. Moreover, our predictions are available for 459 human miRNAs and a variety of cell conditions from our bicluster database, called BiMIR (http://btool.org/bimir_dir/). We further validated our approach by analyzing the pathways of cancer-specific biclusters and prognosis of associated miRNAs followed by confirmatory experiments.

## MATERIALS AND METHODS

### Collection of expression fold-change data

We downloaded CEL files for 2019 GEO series produced using the Affymetrix U133 Plus 2.0 chip. Robust multi-array average (RMA) normalization was applied to each CEL file using ‘justRMA’ function in R ‘affy’ package (32). The intensities of probes for each gene were collapsed by their average value. Next, we curated two sample groups (test/control) for each experimental series and calculated the logarithmic FC (denoted as logFC) of the average expressions in each group. In total, logFC profiles for 5158 (test/control) cell conditions were collected for 20 639 human gene symbols. The logFC matrix and information of the cell conditions are available from our bimir R package (https://github.com/unistbig/bimir).

### Sequence-specific miRNA targets

Sequence-specific miRNA targets were obtained from the seven sequence-based target prediction databases (TargetScan (33), miRanda (34), mirSVR (35), PITA (36), DIANA-microT (37,38), miRDB (39) and TargetRank (40)). The number of candidate miRNA–mRNA interactions, parameters used and download sites for the sequence-specific targets are available in Supplementary Data (Section S1).

### MiRNA target prediction using a progressive bicluster extension (PBE) algorithm

**Figure 2.**
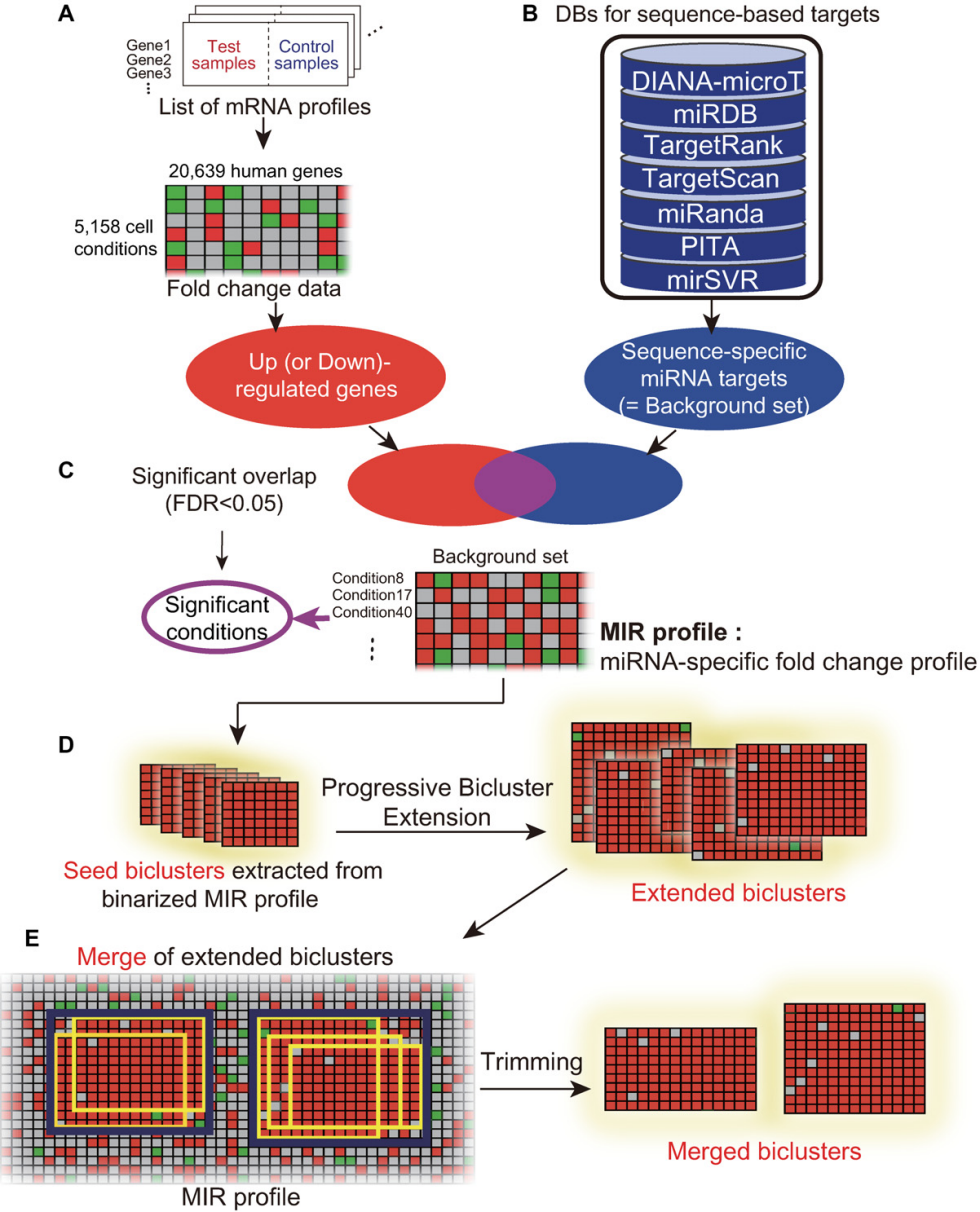
Overview of the biclustering-based miRNA target prediction. (**A**) The gene expression fold-change compendium. (**B**) Sequence-specific targets for each miRNA were obtained from seven miRNA target databases. (**C**) The MIR profile is composed of binarized logarithmic fold-change values of sequence-specific targets for selected cell conditions. (**D**) From MIR profile, seed biclusters are extracted using BIMAX algorithm, and then are extended using PBE algorithm. (**E**) Finally, merged biclusters are generated by hierarchical clustering of extended biclusters and removing the noisy rows and columns.

The overview of biclustering-based miRNA target prediction is shown in Figure 2. First, 5158 mRNA microarray datasets with two sample groups (test/control) were collected from GEO database (24), and corresponding logFC data were compiled for 20 639 human genes (columns) and 5158 fold-change cell conditions (rows). These logFC data are quantized into up-, neutral- and down-regulated genes (denoted as 1, 0 and −1, respectively) using $ \pm$log_2_1.3 (hereafter, simply denoted as 1.3 FC) thresholds. We regarded 1.3 FC as an appropriate threshold for representing target expression changes caused by miRNA regulation excluding noisy data and covering many ‘fine-tuned’ mRNA targets simultaneously. For each miRNA, sequence-specific targets predicted in at least three out of seven miRNA target databases were selected (denoted as background set). Then, logFC profiles for each condition were accumulated to the background set based on the enrichment of 1.3-fold up-regulated genes in the background set (hypergeometric test, FDR < 5%). The resulting logFC submatrix was converted to a binary matrix by replacing −1 with 0, and was dubbed *MIR profile* for the given miRNA. We first applied the bimax biclustering algorithm (27) to the MIR profile to obtain a number of small biclusters completely filled with 1 (called seed biclusters). These seed biclusters were then ‘progressively’ extended using PBE algorithm (extended biclusters); rows and columns with many 1’s were competitively added to the seed bicluster and then relatively noisy rows and columns were removed, and this process was repeated by slightly increasing the threshold for zero proportion in each row and column (strict to less strict). The extended biclusters were then clustered using average-linkage hierarchical clustering (merged bicluster) to remove redundant results. The Meet/Min distance was used for hierarchical clustering as follows: For two different extended biclusters A and B,

\begin{equation*}{\rm{Distance}}\;\left( {{\rm{A}},{\rm{B}}} \right) = 1 - \frac{{\left| {A \cap B} \right|}}{{\min \left( {\left| A \right|,\left| B \right|} \right)}},\end{equation*}

where $| {\rm{A}} |$ is the multiplication of the row and column sizes of A. We tested for the three cutoff values (0.3, 0.5 and 0.7) for the cluster dendrogram. This cutoff had a limited effect on the result, and thus we used the cutoff = 0.5. After the merging, the rows or columns containing more than 10% of zeros were trimmed off individually, finally yielding the ‘merged biclusters’. See Supplementary Data for a detailed description of PBE algorithm (Section S2, Supplementary Figures S1 and S2). Only the merged bicluster was used for target prediction and is simply denoted as ‘bicluster’ hereafter unless noted otherwise.

The resulting biclusters represent predicted target genes (bicluster columns) up-regulated for the clustered cell conditions (bicluster rows). Down-regulated biclusters were also generated in the symmetrical way. Up (down)-regulated biclusters imply that the corresponding miRNA is down (up)-regulated in the captured test conditions. Detailed features of the biclusters are described in Supplementary Data (Supplementary Figure S3 and Supplementary Table S2). We mainly reported the analysis results for 1.3 FC threshold, but biclusters were also generated under $ \pm$log1.5 and $ \pm$log2.0 thresholds (denoted as 1.5 FC and 2.0 FC thresholds, respectively) to capture more specific and stronger miRNA regulation. Overall, for the list of sequence-specific targets of a given miRNA, two MIR profiles (up and down) are generated for each threshold (1.3, 1.5 and 2.0). The three up-regulated (and down-regulated) MIR profiles have different condition counts, while the gene counts are the same. Therefore, the resulting seed bicluster (and the final merged bicluster) counts differ for different thresholds. An example of let-7c bicluster for stem cell conditions are described in Supplementary Data (Section S5).

### Experimental validation of miR-29b/c regulation in breast cancer

#### miRNA transfection

miR-29b-3p and miR-29c-3p mimic and miRNA scramble control were purchased from Genolution. Each miRNA (100 nM) were transiently transfected into MDA-MB-231 by using G-fectin Reagent (Genolution). All experiments were performed 48 h after transfection.

#### Real-time quantitative PCR

One microgram of total RNA from MDA-MB-231 cell was reverse transcribed with oligo dT and M-MLV RT reverse transcriptase (Invitrogen). Real-time quantitative PCR was performed using a GENETBIO SYBR Green Prime Q-master Mix and the QuantStudio 5 PCR system (ThermoFisher). All runs were accompanied by the internal control B2M or HPRT gene. Because both the reference genes yielded very similar results, only B2M results are shown in Figure 6. The samples were run in duplicate and normalized to B2M or GAPDH using a DD cycle threshold-based algorithm, to provide arbitrary units representing relative expression.

#### Immunoblotting

Harvested cells were lysed in RIPA buffer and subjected to centrifugation, and the supernatants were collected. Protein concentration was measured using the BCA protein assay kit (Pierce), and equal amounts of protein were resolved using 10% or 12% sodium dodecyl sulfate (SDS)-polyacrylamide gel electrophoresis (PAGE) and transferred to Nylon membranes (GE Healthcare, Amersham). Target proteins were observed by incubation with primary antibodies and infrared fluorescence dye-conjugated secondary antibodies as follows: rabbit anti-human FAK (1:1000, cell signaling), phospho- FAK (1:1000, cell signaling), Akt (1:1000, cell signaling), phospho- Akt (1:1000, cell signaling) and mouse anti-human GAPDH (1:1000, cell signaling). The HRP-conjugated secondary antibodies were purchased from Cell Signaling Technology. Immunodetection was performed using an Odyssey CLx scanner (Li-COR Biosciences).

## RESULTS

### Comparison with other biclustering algorithms

Compared to seed biclusters, PBE algorithm yielded much larger biclusters by allowing for a small portion of noise (Supplementary Figure S3). Its performance was compared with those of five existing biclustering algorithms such as ISA (28), QUBIC (29), FABIA (30), BiBit (31) and HOCCLUS2 (17). A summary of each method is described in Supplementary Data (Section S4). First, the size and signal density of biclusters generated from a real up-regulated MIR profile of let-7c-5p were compared (Supplementary Table S3). PBE yielded large biclusters with high densities, whereas existing algorithms yielded biclusters with either smaller sizes or poorer densities. PBE also captured stem-cell-specific bicluster better than the other algorithms (Supplementary Figure S4). Detailed results for real data analysis are described in Supplementary Data (Section S4).

**Figure 3.**
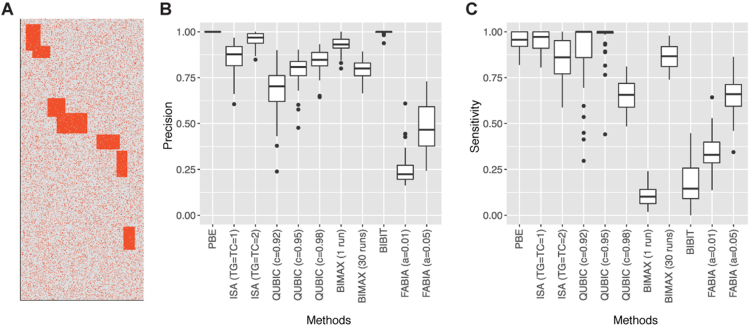
Simulation test for biclustering algorithms. (**A**) Example of simulation profile. Orange and gray elements indicate 1 and 0, respectively. (**B**) Precision and (**C**) sensitivity of tested biclustering algorithms.

Next, we tested the sensitivity and specificity of biclustering algorithms using simulated binary profiles that reflect the average size and density of real MIR profiles (700 rows, 300 columns and 20% density) (Figure 3A). The simulated profiles contained seven biclusters in which row and column sizes were randomly chosen between 20 and 80, and each bicluster included 1–3% of zeros (noise). Some of biclusters overlapped with each other by <20% of the bicluster sizes. The simulation was repeated 50 times. Here, ‘true elements’ indicate those included within the seven biclusters, and ‘false elements’ indicate those outside the biclusters. Thus, after running each biclustering algorithm, the sensitivity was measured as the number of true elements within the predicted biclusters divided by the number of all true elements. The precision was measured as the proportion of true elements within the predicted biclusters. PBE showed perfect precision (median = 100%) with high sensitivity (median = 95.6%). The performance of ISA depended on the row (TG) and column (TC) thresholds. When TG = TC = 1, high sensitivity was observed (median = 97.2%) while precision was relatively low (median = 87.7%). When both TG and TC were increased to 2, the precision was increased (median = 96.8%) but the sensitivity was decreased (median = 86.1%). The QUBIC results were affected by the consistency parameter *c*. As this value was increased, precision was increased while sensitivity was decreased. The best performance was observed when using the default parameter (*c* = 0.95, median precision = 80.8%, median sensitivity = 100%). BIMAX and BiBit do not allow zeros in the biclusters and exhibited quite low sensitivities (median BIMAX sensitivity = 10.2%, median BiBit sensitivity = 14.5%). However, when 30 iterations were applied for BIMAX, its sensitivity was much increased to 86.7%. FABIA yielded highly noisy biclusters for all tested sparseness parameters (*a*) resulting in low precision (median $ \le$ 46.6%) and sensitivity ($ \le {\rm{\ }}$66.0%). Results for *a* = 0.01 and 0.05 are shown in Figure 3. For *a*${\rm{\ }} \ge {\rm{\ }}$0.1, FABIA did not create a bicluster. HOCCLUS2 was also tested but excluded from Figure 3, because it did not generate any bicluster under this simulation setting. HOCCLUS2 detected biclusters from sparser data (12% or lower density). These results indicate that PBE is an efficient algorithm to identify biclusters from noisy binary data.

### Accuracy of the biclustering target prediction

**Figure 4.**
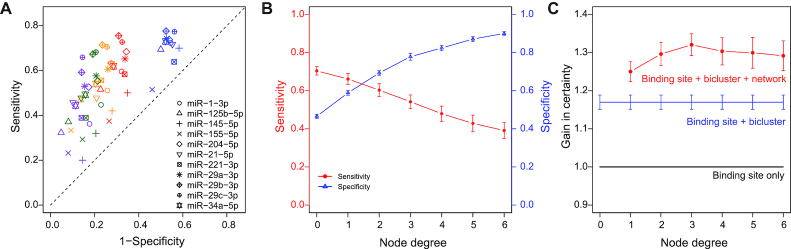
Performance of miRNA target prediction using binding sequence, biclustering and functional networks. (**A**) Sensitivity and specificity of pooled bicluster targets of 11 miRNAs. Targets with binding sequences were used as background (diagonal black dash). Blue nodes represent biclustering results. Red/yellow/green/purple nodes represent the results obtained using both the biclustering and network-based target selection with node degrees 2, 3, 4 and 5, respectively. (**B**) Average sensitivity and specificity for different node degrees of target networks. (**C**) Average gains in certainty of methods using binding sequence, biclustering and network information.

The bicluster targets were assessed using validated miRNA targets. miRTarBase (41) provides hundreds of thousands of experimentally validated miRNA-target relations with ‘strong’ evidence (reporter assays or western blot) and ‘less strong’ (or weak) evidence (pSILAC or microarray experiment). Among the sequence-specific targets (background set) of a given miRNA, those validated with ‘strong’ evidence were regarded as gold positive (GP) targets, whereas those having neither strong nor weak evidence were set as gold negative (GN) targets. For evaluation, we selected miRNAs having more than 30 GPs whose fraction within the background set was not <5%. Eleven miRNAs that satisfied these criteria were analyzed (Figure 4A).

For each miRNA, all the resulting bicluster targets, whether up- or downregulated, were pooled as predicted targets, and corresponding sensitivity, specificity, as well as GP enrichment and GN depletion were calculated (Supplementary Tables S5 and S6). When the 1.3 FC threshold was used to quantize the logFC data, the average sensitivity and specificity of the 11 miRNAs were 0.704 and 0.466, respectively (summation = 1.170), representing a 17.0% (median 19.4%) improved gain compared with the sequence-based target prediction. Although positive gains were obtained for all 11 miRNAs for the 1.3 FC cutoff (Figure 4A), the relative performances for each miRNA were quite different for different FC cutoffs (Supplementary Table S5). For example, the gain of miR-34a-5p decreased as the FC cutoff was increased because of the rapid decline in sensitivity (gains for 1.3 FC: 20.8%, 1.5 FC: 13.3%, 2.0 FC: 7.2%). In contrast, the gain of miR-21-5p increased as the cutoff was increased because the specificity was relatively more increased (gains for 1.3 FC: 16.4%, 1.5 FC: 26.5% and 2.0 FC: 31.3%). Such a difference presumably represents different miRNA regulation patterns. The former case corresponds to the ‘fine tuner’ miRNAs that moderately regulate many genes. Therefore, using a lower cutoff helps detect subtle changes in target expressions. However, miRNAs for the latter case seem to more strongly regulate a relatively small number of targets. Among the three thresholds tested, 1.3 FC exhibited the best overall gain with the largest sensitivity.

miRNA targets tend to be functionally related with each other (42,43). Therefore, we incorporated the protein functional interaction networks from the STRING database (44) (edge threshold > 150) between the bicluster target genes to improve the prediction. Among the bicluster targets, we further selected those with *k* or more functional interactions with other targets and measured the corresponding gains. Intriguingly, the specificity rapidly increased as *k* was increased (Figure 4B), and the maximum gain reached up to 32.0% when *k* = 3 (specificity = 77.8%, Figure 4C). The maximum median gain was even higher (33.4% when *k* = 4). These results show that target interaction networks can improve the miRNA target prediction considerably.

### Comparison with anticorrelation-based methods in cancer

**Figure 5.**
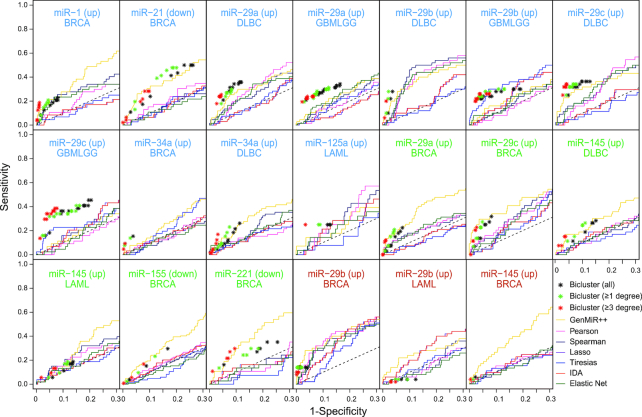
Performance comparison between biclustering and anticorrelation-based methods. Black asterisks represent biclustering predictions. Green and red asterisks represent bicluster targets with at least one and three network degrees, respectively. Solid lines represent ROCs of the seven anticorrelation-based methods. The title of each panel represents the cancer type, miRNA and target regulation direction (parenthesized). Blue, green and red titles represent the 11, 6 and 3 cases where the biclustering method performed better than, similar to and worse than anticorrelation-based methods, respectively. Dashed black lines represent the background results when only sequence-specific targets were used. BRCA, DLBC, GBMLGG and LAML represent breast invasive carcinoma, diffuse large B-cell lymphoma, glioma and acute myeloid lymphoma, respectively.

miRNA–mRNA paired profiling has been commonly used to predict condition-specific miRNA targets based on the anticorrelation between miRNA and its mRNA targets. Therefore, we compared our biclustering method with seven anticorrelation-based methods (GenMiR++(13), Pearson correlation, Spearman correlation, Lasso (45,46), Elastic Net (47), IDA (48) and Tiresias (49)) in predicting cancer-specific miRNA targets. Pearson/Spearman correlation, Lasso, Elastic Net and IDA were implemented using miRLAB R package (50), and GenMiR++ and Tiresias were run using original MATLAB and Perl codes, respectively. For the 11 miRNAs evaluated in the previous section, the accuracy of the predicted targets was compared between anticorrelation-based methods and our biclustering method. For the anticorrelation-based methods, the sequence-specific targets of each miRNA were sorted in the order of anticorrelation scores that were calculated from TCGA (The Cancer Genome Atlas) miRNA–mRNA profiles by Pearson/Spearman correlation, Bayesian method, penalized regression or neural network model. These sorted scores were compared to the gold standard positive/negative sets that yielded ROC curves. For the biclustering method, we selected biclusters where at least 30% of the rows pertained to ‘tumor versus normal’ or ‘aggressive versus non-aggressive tumor’ conditions. These biclusters represented 33 miRNA-cancer pairs for five cancer types (breast, brain, lung, colon or blood cancer). In each miRNA–cancer pair, corresponding bicluster targets were pooled in the order of proportion of the specific cancer condition in each bicluster. Thus, the true and false-positive rates of bicluster targets in each pooling step were depicted instead of ROC curve (asterisks, Figure 5). After removing six cases where none of the areas under ROC curves (AUCs) exceeded 0.6 and the maximum biclustering gain was <1.1, we selected biclusters from 20 cases that were coherent with known miRNA expression (quantitative PCR results) for comparison. In other words, upregulated biclusters were chosen when corresponding miRNA was known to be downregulated and vice versa, in cancer. Supplementary Table S7 lists the literature reporting the expression levels of miRNAs in cancers.

Overall, the biclustering method was compared favorably with the miRNA–mRNA profile based methods (Figure 5). For 11 out of the 20 cases, the biclustering method exhibited better gains than the anticorrelation-based methods; in 6 other cases, both approaches exhibited similar performances; in the remaining 3 cases, the biclustering method was inferior to the best anticorrelation-based method, mostly because of its low sensitivity. As seen in the previous section, incorporating the network information tended to increase the specificity and gain of the biclustering method. Among the seven anticorrelation-based methods, Genmir++ performed best for most cases.

These results showed that if miRNA expression information was provided, our biclustering approach overall performed better than anticorrelation-based methods in prioritizing condition-specific miRNA targets. Notably, miRNA expression is relatively easily obtained from the literature or quantitative PCR experiments.

### miRNAs targeting PI3K/Akt signaling in cancer

We further analyzed the bicluster targets corresponding to the 20 cancer-miRNA pairs (Figure 5). Among them, breast cancer and diffuse large B-cell lymphoma (DLBCL) yielded the largest numbers of biclusters. In breast cancer, bicluster targets of miR-1, miR-29a/b/c, miR-34a and miR-145 were upregulated in aggressive cancer; in DLBCL, the targets of miR-29a/b/c, miR-34a and miR-145 were also upregulated. We pooled those bicluster targets in each cancer type and performed pathway enrichment analysis (KEGG annotation) using the DAVID tool (51) to identify seven and four significant pathways (FDR < 0.05) in breast cancer and DLBCL, respectively (Supplementary Tables S8 and S9). Interestingly, the bicluster targets in both cancer types were strongly enriched with ‘PI3K/Akt signaling pathway’ (FDR = 2.6E-7 for breast cancer; FDR = 5.3E-7 for DLBCL). This pathway is known to be frequently hyperactivated in many cancers to promote cell cycle and survival, proliferation and epithelial–mesenchymal transition of tumor cells (52,53). In addition, extracellular matrix (ECM)–receptor interaction and focal adhesion pathways were commonly caught in both cancer types, but all the corresponding bicluster targets except two (CAV2, BIRC2) were also included in PI3K/Akt signaling pathway.

**Figure 6.**
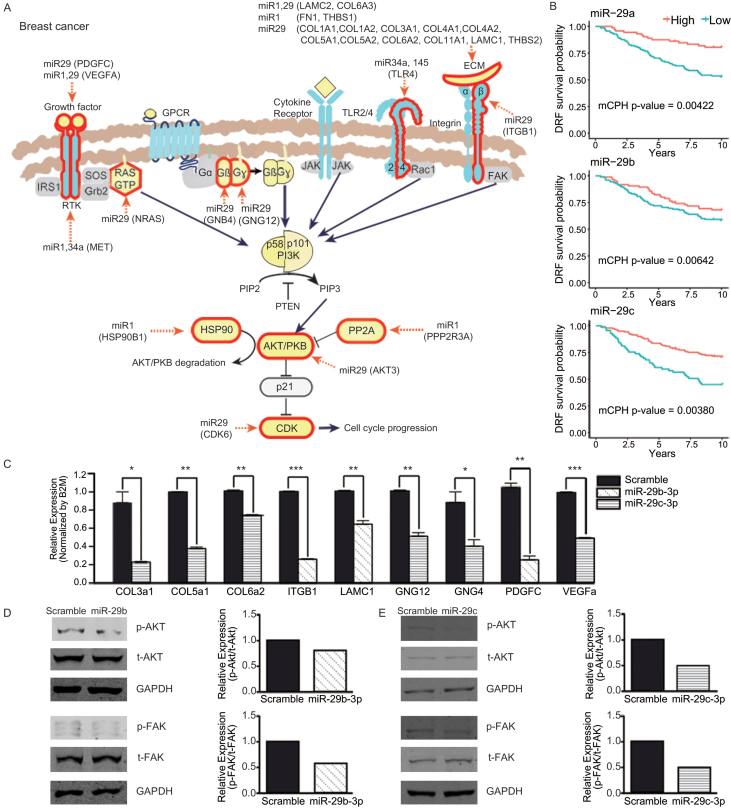
miRNA targets in PI3K/Akt signaling pathway (breast cancer). (**A**) miRNA targets predicted from breast cancer biclusters are highlighted by red borders. For each target molecule, corresponding miRNA names and target gene symbols are represented. (**B**) Distant relapse-free survival analysis for 210 patients with breast cancer exhibiting high and low miR-29a, miR-29b and miR-29c levels. The patients were divided into two groups based on their best splits at top 33.8%, 40% and 66% values, respectively. (**C**) Transcript levels of miR-29 target gene candidates were analyzed by qRT-PCR. MDA-MB-231 breast cancer cells were transiently transfected with either scrambled miRNA (control) or miR-29 (29b-3p or 29c-3p). All the nine genes tested were considerably downregulated by miR-29b and/or -29c. In particular, ITGB1, GNG12 and VEGFA were downregulated by both miR-29b and -29c. Statistical significance was tested by one-tailed *t*-test. *P < 0.05; **P < 0.01; ***P < 0.001 versus scrambled miRNA. (**D** and **E**) Activation of downstream pathway candidates such as AKT and FAK were analyzed by immunoblotting. Total cell lysates extracted from either scrambled miRNA or (D) miR-29b-3p as well as (E) miR-29c-3p transfected cells were analyzed for the levels of pAKT, AKT, pFAK and FAK.

Figure 6A and Supplementary Figure S5A show PI3K/Akt pathway where the bicluster targets are highlighted for breast cancer and DLBCL, respectively. In both cancer types, the miRNAs targeted multiple ligands including genes encoding growth factors (e.g. VEGFA and PDGFC targeted by miR-29) and ECM (e.g. COL1A1, LAMC1 and THBS2 by miR-29); signal transducers such as receptor tyrosine kinase (e.g. MEK and/or PDGFRA by miR-34a), G-proteins (GNB4 and GNG12 by miR-29), toll-like receptor (TLR4 by miR-34a and miR-145) and integrin (e.g. ITGB1 by miR-29); as well as downstream effectors such as NRAS (by miR-29 and miR-145) and CDK6 (by miR-29). In addition, AKT3 was targeted by miR-29 in breast cancer, and cytokine receptor (IL2RB and IL6R) and one component of the PI3K complex (PIK3R3) were also targeted by miR-34a and miR-29, respectively, in DLBCL. Indeed, it was previously shown that miR-29b upregulation in breast cancer considerably inhibited metastasis by repressing targets related to the tumor microenvironment (54) (including some genes listed above).

In the present study, we experimentally validated the bicluster targets of miR-29 using the human breast cancer cell line, MDA-MB-231, which is a well-established metastatic and invasive cancer cell line. Transcript levels of nine bicluster targets related to ECM or PI3K were analyzed 2 days after transient transfection with either miR-29 or control miRNA. All the nine targets were significantly downregulated by miR-29b or -29c transfection compared to the controls (Figure 6C). Furthermore, the activation of ECM-related downstream pathways such as focal adhesion kinase (FAK) and AKT were also attenuated by miR-29 (Figure 6D and E) demonstrating the capability of biclustering analysis to capture relevant pathways for disease.

Finally, we analyzed the prognostic values of these miRNAs using multivariate Cox proportion hazard (mCPH) model for public miRNA expression datasets. The distant-relapse-free survival was tested for 210 patients with breast cancer (GEO database, GSE22216). Among the six miRNAs analyzed, the three miR-29 family miRNAs had significant prognostic values (mCPH *P*-values of miR-29a = 0.0042, miR-29b = 0.0064, miR-29c = 0.0038; adjusted for age, tumor size, lymph nodes involved, ER and grade). Then, the overall survival of 116 patients with DLBCL (GSE40239) was also analyzed for five miRNAs. Among them, two exhibited significant prognostic values (mCPH *P*-values for miR-34a = 0.0185 and miR-145 = 0.0041; adjusted for International Prognostic Index (IPI) and gender). See Supplementary Tables S10 and S11 for detailed results. Kaplan–Meier plots contrasting the effects of miRNA expression on survival are also shown in Figure 6B and Supplementary Figures S5B and S5C.

Overall, by analyzing cancer biclusters, we were able to identify the key pathways (PI3K/Akt signaling, ECM and focal adhesion), and five associated prognostic miRNAs (miR-29a, miR-29b and miR-29c in breast cancer; miR-34a and miR-145 in DLBCL) that are repressive of tumor progression (hazard ratios of 0.593–0.745). In particular, the effects of miR-29b/c on these pathways were validated experimentally (Figure 6C-E).

### BiMIR: a bicluster database for condition-specific miRNA targets

In total, 29 898 biclusters were generated for 459 human miRNAs using PBE algorithm (13 949 for 1.3 FC; 10 999 for 1.5 FC; 4950 for 2.0 FC thresholds) and compiled in BiMIR database (http://www.btool.org/bimir_dir/) where biclusters are searchable for miRNAs, tissues, diseases, keywords, target genes of interest and their combinations. BiMIR can be used for investigating novel miRNA functions, targets and related cell conditions.

Along with the list of searched biclusters, the function enrichment results for bicluster targets are provided based on the MSigDB (55) pathway (C2) and gene ontology (C5) categories. If biclusters are searched for a specific organ/tissue or disease, the proportion of corresponding conditions in each bicluster is also indicated. These help the user find relevant biclusters. The heat maps for each bicluster are visualized (Supplementary Figure S6) and corresponding target genes and cell conditions are hyperlinked to Genecards (56) and GEO (24) databases for detailed information, respectively. For bicluster target genes, the experimental evidence from miRTarBase (41), network node degrees and protein network visualization based on STRING database (44) are provided. All the biclusters are downloadable from BiMIR database.

## DISCUSSION

Here, we presented a novel framework to prioritize miRNA targets by biclustering sequence-specific targets and cell conditions, which is a dimension that has been rarely investigated. This is based on the idea that miRNA targets, like other cellular molecules, have modular activity and can be repeatedly captured across different cell conditions. Indeed, the bicluster targets exhibited substantially improved accuracy compared to purely sequence-based targets and were often enriched in well-known pathways characterizing the modules identified. Moreover, functionally connected targets exhibited even higher accuracy, further confirming the modular activity of miRNA targets. The functional interaction of miRNA targets and their contribution to target prediction have been studied previously (57,58).

We analyzed cancer biclusters and found that PI3K/Akt signaling pathway was intensively targeted by a few miRNAs in two cancer types. Further, prognostic values of those miRNAs and the regulatory effects of miR-29 were also validated. These results demonstrate that biclustering analysis is able to reveal key pathways controlled by miRNAs in disease. BiMIR database provides miRNAs and targeted pathways for dozens of diseases.

Based on the knowledge of miRNA expression, our prediction was favorably compared with seven anticorrelation-based methods under cancer conditions. These results demonstrate the practical value of our approach in that our results can provide fairly good target predictions for a variety of cell conditions without generating costly miRNA–mRNA profiles. BiMIR database was designed to explore the modular regulatory networks of miRNAs by connecting miRNAs, cell conditions (or disease), mRNA targets and associated pathways. The user may obtain candidate miRNAs and target genes for a cell condition of interest. Knowledge of the miRNA expression level will help select the proper direction of biclusters (up or down).

Despite the improvements and usefulness shown in this study, there remain difficulties in our approach regarding free parameters that need to be optimized. First, the minimum seed size of 10 × 10 was determined in an *ad hoc* manner, and its optimal size may be affected by the size of the fold-change data. Second, the iteration number of 20 in BIMAX algorithm was used to compromise the computation time, using a higher iteration number yielded more biclusters. However, other parameters seemed to be less sensitive. For example, we gradually increased the threshold of zero proportion from 0.01 to 0.1 (step size 0.01) during 10 iterations of bicluster extension. This may seem to allow 10% of zeros in the end, but the final zero proportion was only ∼1.5% because of the trimming process. The cutoff of hierarchical clustering of the extended clusters was also a less sensitive parameter. In addition, the biclusters were generated under a rather strict criterion (for targets in three or more databases); therefore, BiMIR can be used for selecting a small number of highly likely targets for the cell condition of interest.

The biclustering approach presented here can also be applied for predicting the condition-specific targets of other sequence-specific regulators such as transcription factors or RNA-binding proteins. In this regard, the entire 5158 mRNA fold-change profiles for 20 639 genes are provided for general systems biology research. These mRNA fold-change data are different from the GTEx transcriptome data (59) in that GTEx data represent transcription levels in normal tissues, whereas our fold-change data represent gene expression ‘changes’ for a variety of cell conditions such as disease, chemical treatment, tissues and differentiations. Thus, these fold-change data can also be used for clustering or regulatory network analysis for a specific group of genes or cell conditions.

Whereas existing methods to identify miRNA regulation modules bicluster multiple miRNAs and multiple target genes representing coregulatory networks, our work presented here is focused on prioritizing highly likely target genes of a single miRNA that are commonly detected across multiple cell conditions. Our approach can also be extended to evaluate the miRNA coregulatory networks by overlapping biclusters for different miRNAs. A significant overlap indicates mRNA targets coregulated under multiple cell conditions. Our approach and data would contribute to uncovering the modular structure of complex regulatory networks.

## DATA AVAILABILITY

BiMIR database are available at http://www.btool.org/bimir_dir/. BiMIR R package that includes the biclustering code and the large expression fold-change data are available at https://github.com/unistbig/bimir.

## Supplementary Material

Supplementary Data
