# Supplementary material for: Biclustering analysis of transcriptome big data identifies condition-specific microRNA targets
Source: Nucleic Acids Res. 2019 Mar 1;47(9):e53. doi: 10.1093/nar/gkz139 (PMC6511842; doi:10.1093/nar/gkz139)
Supplement: Supplementary Data [file gkz139_supplemental_files.zip › bimir_Suppl.docx]

**Supplementary Data**

***%% Table S5, S6, S8, and S9 are available from separate excel files.***

**Table S1. miRNA target prediction methods**

| **Sequence-based methods** | | |
| --- | --- | --- |
| **Method** | **Features** | **Ref.** |
| TargetScan | Seed match, Conservation | (1) |
| PITA | Seed match, Conservation, Free energy, Site accessibility, Target-site abundance | (2) |
| miRDB | Seed match, Conservation, Free energy, Machine learning | (3) |
| mirSVR | Seed match, Conservation, Free energy, Site accessibility, Machine learning | (4) |
| miRanda | Seed match, Conservation, Free energy | (5) |
| DIANA-microT-CDS | Seed match, Conservation, Free energy, site accessibility, Target-site abundance, Machine learning | (6,7) |
| TargetRank | Seed match, Conservation, Base composition at position t9, flanking AU content | (8) |
| **Correlation/Causality-based methods** | | |
| **Method** | **Features** | **Ref.** |
| Pearson correlation | Pearson correlation between an mRNA and miRNA | (9) |
| Spearman correlation | Spearman correlation between an mRNA and miRNA | (10) |
| Lasso | Lasso regression coefficient between an mRNA and miRNA | (11,12) |
| ElasticNet | ElasticNet regression coefficient between an mRNA and miRNA | (13) |
| GenMIR++ | Bayesian learning algorithm | (14) |
| Tiresias | Two-stage artificial neural network | (15) |
| IDA | Causal structure learning and causal inference | (16) |
| **Biclustering(module)-based methods** | | |
| **Method** | **Features** | **Ref.** |
| BIMIR | Biclustering mRNA targets and cell conditions using large log expression fold change data | - |
| Yoon and De Micheli, HOCCLUS2 | Biclustering mRNA and miRNA using mRNA:miRNA interaction score matrix | (17,18) |
| Peng et al., Joung et al., Liu et al., Jayaswal et al. | Biclustering mRNA and miRNA using target sequence and mRNA:miRNA paired expression data | (19-22) |

**S1. Collection of sequence-based miRNA targets**

The sequence-based miRNA targets were set as those predicted from three or more miRNA target prediction databases listed below.

- TargetScan (version 7.0): TargetScan data (Conserved site context++ scores) provided 253,132 miRNA-target interaction data. It was downloaded from TargetScan homepage (<http://www.targetscan.org> ).
- PITA (version 6): PITA data (PITA_targets_hg18_0_0_ALL.txt) provided 4,095,751 miRNA-target interaction data. Among them we used 716,486 interactions of which free energy scores were less than -10. The data was downloaded from <https://genie.weizmann.ac.il/pubs/mir07/mir07_data.html>.
- miRDB (version 5.0): miRDB data (miRDB_v5.0_prediction_result.txt) provides 1,873,265 miRNA-mRNA interaction data. Among them we used 1,314,352 interactions of which scores were greater than 60. The data was downloaded from <http://www.mirdb.org/download.html>.
- MicroCosm Targets: MicroCosm Targets provided 728,288 miRNA-target interactions. The data was downloaded from <http://www.ebi.ac.uk/enright-srv/microcosm/htdocs/targets/> but not available now.
- miRanda (microRNA.org): miRanda provided 1,097,064 conserved miRNA-mRNA interactions with high mirSVR score (human_prediction_S_C_aug2010.txt). The data was downloaded from <http://www.microrna.org/microrna/getDownloads.do> but not avaliable now.
- DIANA-microT-CDS (version 5.0): DIANA-microT-CDS provides 7,337,705 miRNA-mRNA interactions. Among them we used 1,457,011 interactions of which scores were larger than 0.7.
- TargetRank: TargetRank provides 1,006,494 miRNA-mRNA interactions. The data was downloaded from (<http://hollywood.mit.edu/targetrank/hsa_miRBase_miR_ranked_targets.txt> ).

**S2. Progressive Bicluster Extension (PBE) algorithm**

The overall process of PBE algorithm is shown in Figure S1. PBE algorithm is composed of the iteration of two parts: the extension step and the trimming step. Briefly, the seed bicluster is extended by adding the background rows or columns that have the minimum zero rate (extension step) and then noisy rows and columns (showing high zero rate) of the extended bicluster are removed (trimming step). This two-step process is applied *R* times, and the bicluster is updated *R* times accordingly (in this study, R = 10). We set the final maximum zero ratio ($Z_{cut}$) as 10%, because a bicluster with more than 10% zeros may not be regarded a high quality bicluster. This 10% (i.e., 0.1) was divided into 10, and during the R = 10 rounds of bicluster extension, the maximum allowed zero ratio was gradually increased from 0.01 (R = 1) to 0.1 (R = 10) with a small step size 0.01. If the step size is 0.01, we run the iteration R = 10 times. If 0.02, we use R = 5; however, smaller step size will yield tighter (= denser) biclusters. Although 10% final zero ratio was used for the extended bicluster, the actual zero ratio was only 1.5% on average because of the trimming step applied in each iteration of bicluster extension.

***Extension step*.** In the *s*^th^ step of extension ($s=1, 2,\cdots,R$), the intermediate zero rate (Z_cut,s_) allowed in extending the current bicluster is defined as:

$$Z_{cut,s}= \frac{Z_{cut}}{R}\times s$$

For example, if $Z_{cut}$= 0.1 and a seed bicluster is extended through *R* = 10 steps, the $Z_{cut,s}$ for the first extension step will be 0.1*1/10 = 0.01. In other words, stricter criteria are applied in earlier iterations to obtain biclusters with high densities. Let $M$ be the matrix of the MIR profiles, and $R(s)$ and $C(s)$ be the indexes of the rows and columns of the bicluster where s^th^ extension step is done, respectively. $M\left[ R\left( 0 \right), C\left( 0 \right) \right]$ denotes the seed bicluster. After calculating the zero rates in every column vector in $M\left[ R(s-1), {C(s-1)}^{C} \right]$ and row vector in $M\left[ {R(s-1)}^{C}, C(s-1) \right]$, the rows or columns with the minimum zero rate are added to the current bicluster. The same extension process is repeated until the zero rate reaches $Z_{cut,s}$, when the bicluster enters the trimming step.

***Trimming step*.** If any row or column vector with the maximum zero rate exceeds $Z_{cut,s}$, such vector is removed from the bicluster one by one resulting in the updated bicluster $M\left[ R\left( s \right), C\left( s \right) \right]$.

***Prevention of lengthening out in one direction*.** Some biclusters tend to keep lengthening out in one direction if one side of the bicluster becomes too small compared with the other side during the extension process. To ameliorate this, a penalty is given to the longer side if it is more than twice longer than the other side. When the row and column vectors outside the bicluster compete with each other, the following modified zero rate is applied for the longer side vectors.

$$modified zero rate= \frac{\# zeros in the vector+floor(r)}{length of the vector}$$

where,

$$r=\frac{length of longer side of bicluster}{length of shorter side of bicluster}$$

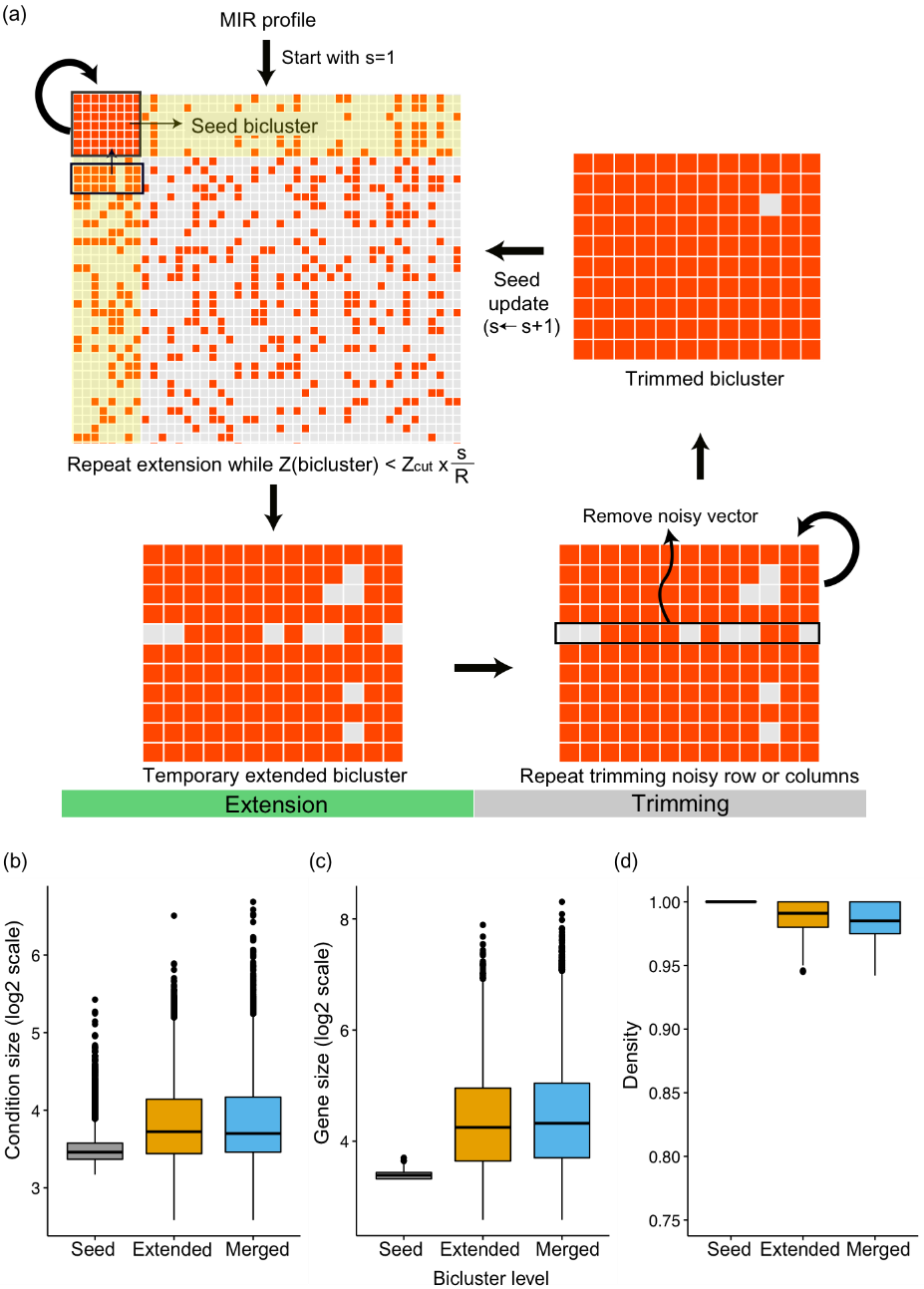


**Figure S1**. Progressive bicluster extension (PBE) algorithm. The final zero rate cut off (Z_cut_) in the extended bicluster should be determined in advance. In the MIR profile, the orange and the grey cells represent 1 and 0 respectively, and the seed bicluster is represented in the black box. The seed bicluster is extended by repeating the following extension and trimming process *R* times. (Extension step) Among the addable vectors (in the yellow shadow), those with the minimum zero rate are simultaneously attached to the current bicluster. If the zero rate in the extended bicluster is less than intermediate zero rate cut-off (Z_cut,s_=Z_cut_*s/R, s means s^th^ repetition step), the extension process is repeated. (Trimming step) If the zero rate exceeds the Z_cut,s_, the rows and columns whose zero rate is larger than Z_cut,s_ are searched for and removed from the most noisy vectors to yield the updated bicluster. The updated bicluster enters next extension/trimming step with updated parameter.

| Input: *V_r_* (condition), *V_c_* (target gene); *M^nxm^* (MIR profile); parameters *S* (The number of extension steps), *Z* (Final zero rate allowed in the bicluster) |
| --- |
| for (i=1 to S) do  $\boldsymbol{Z}_{\boldsymbol{temp}}\boldsymbol{=}\boldsymbol{(Z}/\boldsymbol{S)}\boldsymbol{\times i}$  $\boldsymbol{SEED\leftarrow M[}\mathbf{V}_{\mathbf{r}}\boldsymbol{][}\mathbf{V}_{\mathbf{c}}\boldsymbol{]}$  *# Seed extension process*  *function zero_ratio(array A)*  *return (The # of zeros in A) / \|A\|*  *end function*  *function modified_zero_ratio(array A, integer N)*  *return (The # of zeros in A + N) / \|A\|*  *end function*  *while (zero ratio of SEED <* $\boldsymbol{Z}_{\boldsymbol{temp}}$*) do*  *V_r_* $\boldsymbol{\leftarrow}$ *Conditions in seed bicluster*  *V_c_* $\boldsymbol{\leftarrow}$ *Target genes in seed bicluster*  $\boldsymbol{n}_{\mathbf{1}}\boldsymbol{\leftarrow}\left\vert\mathbf{V}_{\mathbf{r}} \right\vert\boldsymbol{/}\left\vert\mathbf{V}_{\mathbf{c}} \right\vert$ *;* $\boldsymbol{n}_{\mathbf{2}}\boldsymbol{\leftarrow1/}\boldsymbol{n}_{\mathbf{1}}$  *if (*$\boldsymbol{n}_{\mathbf{1}}$*>= 2) then*  *Row_zero* $\boldsymbol{\leftarrow}$ *Values from modified_zero_ratio() for all row vectors in SEED with N=n*  *else*  *Row_zero* $\boldsymbol{\leftarrow}$ *Values from zero_ratio() for all row vectors in SEED*  *end if*  *If (*$\boldsymbol{n}_{\mathbf{2}}$*>= 2) then*  *Col_zero* $\boldsymbol{\leftarrow}$ *Values from modified_zero_ratio() for all column vectors in SEED with N=n*  *else*  *Col_zero* $\boldsymbol{\leftarrow}$ *Values from zero_ratio() for all column vectors in SEED*  *end if*  *Min_row_zero* $\boldsymbol{\leftarrow}$ *minimum of Row_zero*  *Min_col_zero* $\boldsymbol{\leftarrow}$ *minimum of Col_zero*  *New_conditions* $\boldsymbol{\leftarrow}$ *conditions (rows) of M that corresponds to Row_zero==Min_row_zero*  *New_target_genes* $\boldsymbol{\leftarrow}$ *Targets (columns) of M that corresponds to Col_zero==Min_col_zero*  *L1 = \|New_conditions\|; L2 = \|New_target_genes\|*  *if((Min_row_zero < Min_col_zero) OR (MIN_row_zero==Min_col_zero AND L1>=L2)) then*  *SEED_temp_*$\boldsymbol{\leftarrow}$ *M[*$\mathbf{V}_{\mathbf{r}}\boldsymbol{\cup}$*New_condition][ [*$\mathbf{V}_{\mathbf{c}}$*]*  *else*  *SEED_temp_* $\boldsymbol{\leftarrow}$ *M[*$\mathbf{V}_{\mathbf{r}}$*][ [*$\mathbf{V}_{\mathbf{c}}\boldsymbol{\cup}$*New_target_genes]*  *end if*  *if(zero ratio of SEED_temp_ <* $\boldsymbol{Z}_{\boldsymbol{temp}}$*) then*  *SEED* $\boldsymbol{\leftarrow}$ *SEED_temp_*  $\mathbf{V}_{\mathbf{r}}{\boldsymbol{\leftarrow}\mathbf{V}}_{\mathbf{r}}\boldsymbol{\cup}$ *New_conditions*  $\mathbf{V}_{\mathbf{c}}{\boldsymbol{\leftarrow}\mathbf{V}}_{\mathbf{c}}\boldsymbol{\cup}$ *New_target_genes*  *end while*  *# Bicluster Trimming Process*  *Row_zero* $\boldsymbol{\leftarrow}$ *Values from zero_ratio() for all row vectors in SEED*  *Col_zero* $\boldsymbol{\leftarrow}$ *Values from zero_ratio() for all column vectors in SEED*  *Max_row_zero* $\boldsymbol{\leftarrow}$ *maximum of Row_zero*  *Max_col_zero* $\boldsymbol{\leftarrow}$ *maximum of Col_zero*  *While (max_row_zero>*$\boldsymbol{Z}_{\boldsymbol{temp}}$*OR max_col_zero>*$\boldsymbol{Z}_{\boldsymbol{temp}}$*) do*  *if (max_row_zero >= max_col_zero) then*  *conditions_to_delete = SEED conditions (rows) whose zero ratios are equal to max_row_zero*  *V_r_* $\boldsymbol{\leftarrow}$ *V_r_ – Conditions_to_delete*  *SEED* $\boldsymbol{\leftarrow}$*SEED[V_r_ ][*$\mathbf{V}_{\mathbf{c}}$*]*  *else*  *targets_to_delete = SEED target genes (columns) whose zero ratios are equal to max_col_zero*  *V_c_* $\boldsymbol{\leftarrow}$ *V_c_ – targets_to_delete*  *SEED* $\boldsymbol{\leftarrow}$ *SEED[V_r_ ][*$\mathbf{V}_{\mathbf{c}}$*]*  *end if*  *Row_zero* $\boldsymbol{\leftarrow}$ *Values from zero_ratio() for all row vectors in SEED*  *Col_zero* $\boldsymbol{\leftarrow}$ *Values from zero_ratio() for all column vectors in SEED*  *Max_row_zero* $\boldsymbol{\leftarrow}$ *maximum of Row_zero*  *Max_col_zero* $\boldsymbol{\leftarrow}$ *maximum of Col_zero*  *end while*  end for  Return SEED |

**Figure S2**. Pseudocode of progressive bicluster extension algorithm

**S3. Bicluster statistics**

By progressively extending the seed biclusters and merging similar ones, many of missing associations can be restored yielding better biologically results. Figure S3 represents the distributions of bicluster size and density. For 1.3-fold cutoff bicluster, 11.5 conditions and 10.5 genes were included in the seed biclusters on average. After extending them, the average number of conditions and genes were increased to 19.4 and 28.4, respectively. Finally, merged biclusters had slightly more increased sizes. However, the zero ratio of the merged biclusters was only less than 1.5% on average. Increasing FC cutoff resulted in less extended but slightly denser biclusters (Fig S3). Compared with other biclustering methods, PBE was able to identify larger and/or cleaner biclusters from noisy data as shown in the next two sections.

BiMIR (<http://btool.org/bimir_dir/>) provides 29,898 biclusters for 459 human microRNAs. These biclusters cover in total 2,259 fold change (FC) conditions (~43% of total cell conditions). Table S2 shows six statistics of BiMIR biclusters for three binarization cutoffs (1.3, 1.5 and 2.0 FC). Note that for each miRNA, six MIR profiles were generated (up- and down-regulated profiles for three FC cutoffs). If no biclusters were generated from MIR profile, corresponding miRNA was not counted in Table S2.


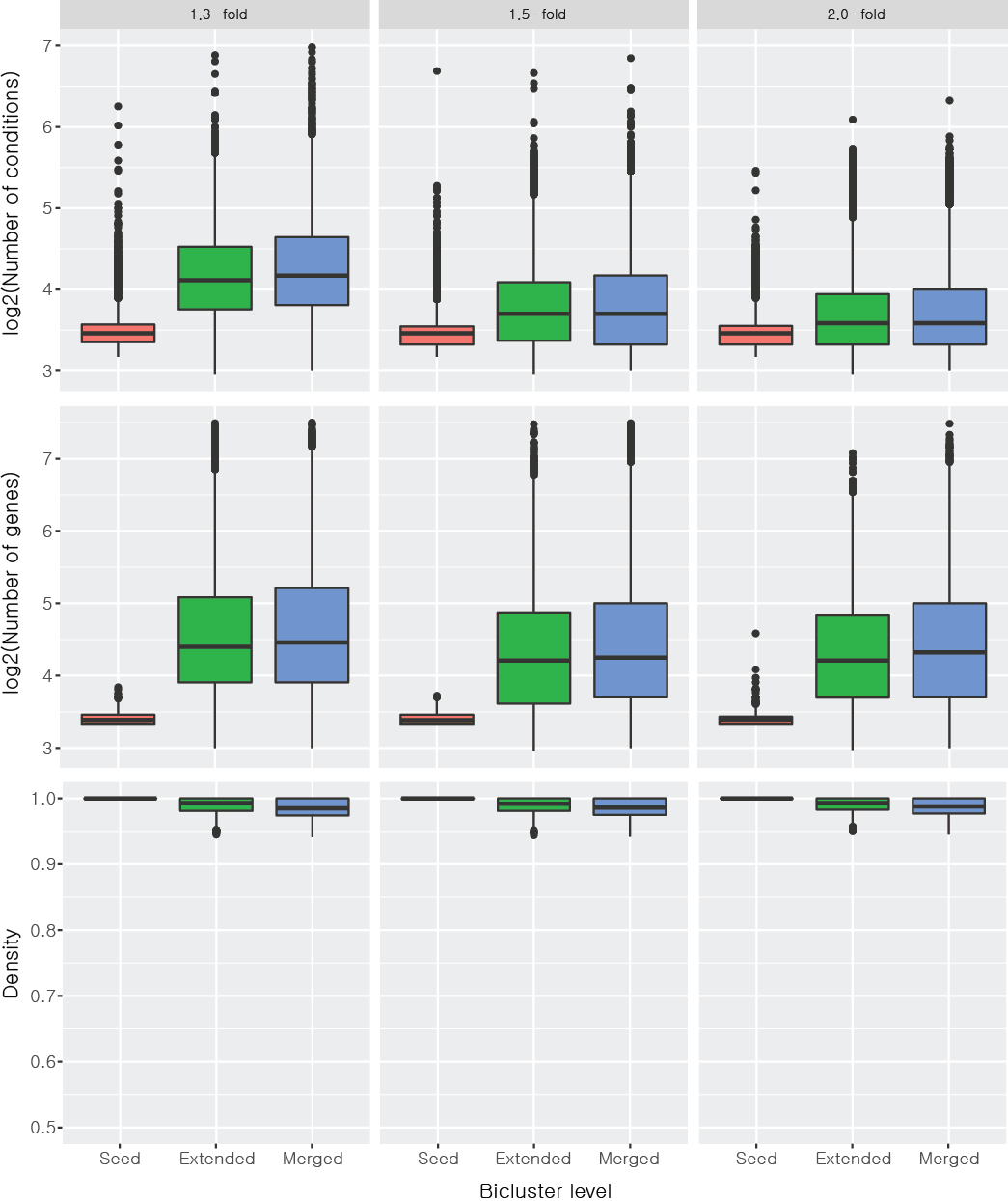


**Figure S3.** Distribution of the number of conditions, genes and density in biclusters with three different fold-change cut-offs.

**Table S2. Statistics of BiMIR biclusters.**

| Binarization cut-off | 1.3 FC | 1.5 FC | 2.0 FC |
| --- | --- | --- | --- |
| Number of miRNAs | 459 | 414 | 348 |
| Number of biclusters | 13,949 | 10,999 | 4,950 |
| Number of FC conditions | 2,259 | 1,828 | 1,057 |
| Average number of conditions | 20.5 | 15.3 | 14.1 |
| Average number of genes | 30.8 | 26.7 | 26.8 |
| Average bicluster density | 0.985 | 0.986 | 0.988 |

**S4. Comparison of biclustering algorithms**

The performance of PBE and other constant biclustering algorithms were compared in two ways: (1) comparison of the size and density of the stem cell related biclusters using MIR profile of hsa-let-7c-5p and (2) comparison of precision and sensitivity using simulated data. In this section, we describe the tested biclustering algorithms, and the comparison results for the stem cell related biclusters. The simulation methods were described in the main text.

1. Compared biclustering algorithms and their implementations for MIR profile of hsa-let-7c-5p

- Iterative signature algorithm (ISA) (23): It was developed to find transcriptional modules from microarray gene expression profiles. It aims to detect a set of genes showing similar up- or down-regulation patterns across a set of samples. To achieve the modules, ISA iteratively updates the rows (genes) and columns (conditions) that satisfy the criterion until the result converges. ISA has two parameters: row (T_G_) and column (T_C_) thresholds. These parameters are set to be 1.0, 1.5, 2.0, 2.5 or 3.0 It was run by ‘isa’ function in ‘isa2’ R package
- QUBIC (24): It is a qualitative or semi-quantitative biclustering algorithm. It automatically converts the continuous input gene expression matrix into signed integer matrix based on the parameters r (e.g., 1: up-regulated, 0: not regulated and -1: down-regulated) and then constructs the gene network in which the edges represent the number of co-regulated conditions. It finds non-overlapping seed biclusters from this network and expand the biclusters based on the consistency parameter that controls the ratio of identical non-zero values in each column. In this study, QUBIC biclusters were generated using BCQUD function in QUBIC R package with three consistency levels (c=0.92, 0.95 and 0.98).
- FABIA (25): It is a generative multiplicative model designed for gene expression data considering the heavy tails in the distribution. It returns biclusters with ranks evaluated according to the information content. FABIA biclusters were generated using ‘fabia’ function in fabia R package for the sparseness loading parameters 0.01, 0.05, 0.1, 0.15, 0.2, 0.25 or 0.3. Parameter p (the number of bicluster) was set as 30 for real data analysis and 7 for simulation data.
- BiBit (26): it was developed for biclustering of binary matrix. It transforms input binary matrix to integer matrix by dividing every rows into bit words of same size and then converting each bit word into decimal number. It is fast by searching for biclusters from this reduced integer matrix. It was run using ParBiBit program (27) which accelerated the running time of BiBit algorithm by implementing MPI parallel programming.
- HOCCLUS2 (17): it was developed to bicluster microRNAs and target genes on binary data (experimentally validated or predicted interaction networks). In the first step, the initial bicliques are generated based on the minimum interaction score. Then, the overlapping biclusters are progressively merged based on the cohesiveness parameter which measures the quality of each bicluster by the functional similarity of genes. HOCCLUS2 has two input parameters α (a cohesiveness threshold) and β (a minimum interaction score). Because 0<β<1 does not affect the result when applied to a binary data, it was fixed to 0.5 and only α was changed from 0.4 to 0.9.

1. Real data analysis

The up-regulated MIR profile of hsa-let-7c-5p (LFC cutoff = log(1.3); 1,526 conditions x 801 genes) was used to compare the performance of different algorithms. Table S3 shows the average row/column size, density, and the number of detected biclusters of each algorithm. PBE identified 17 biclusters having average sizes of 23.3 conditions and 38.9 genes and 98.1% density. ISA was applied to continuous LFC data, and the resulting biclusters were then binarized to estimate their densities for log(1.3) threshold. It is also possible to apply ISA to binarized MIR profile directly. Both ISA results were comparable to each other (Table S3). ISA generated smaller and denser biclusters as larger T_C_ and T_G_ were used. For example, when both parameters were set as 1, the average numbers of conditions and genes were as large as 174 and 119, respectively, but the average density was quite low (50.0%). When both parameters were set as 3, the average density increased to 80.8%, but the average size was quite small (26.1 conditions and 9.2 gene). The average size of BiMIR bicluster was between those of ISA (continuous) biclusters with parameters T_C_ = 2.5, T_G_ = 1.5 - 2.0. In that case, however, the average density of ISA was much lower than that of PBE (70.3% - 71.9%). When the same parameters were applied to binary data, it usually generated larger but sparser biclusters than those obtained from continuous profile. QUBIC was implemented for three consistency parameter values (c = 0.98, 0.95, and 0.92). When c = 0.92, the QUBIC biclusters had fairly large sizes and quite high densities (row size = 15.2, column size = 61.3, density = 97.96%). It tended to contain lesser rows and more columns compared to PBE. This may be caused by the consistency criterion (the minimum ratio of 1 in each column) which makes it difficult to extend row numbers. In addition, QUBIC biclusters sometimes contain highly noisy rows (See the bottom row in Figure S4b). FABIA was also tested for both continuous and binarized MIR profiles using various sparseness parameters (alpha = 0.01, 0.05, 0.1, 0.15, 0.2, 0.25, 0.3). The continuous biclusters were quite sparse for all conditions (density = 31.8% - 38.8%). The binarized biclusters showed higher densities compared to those of continuous biclusters (35.8% - 72.3%), but they were still sparser than those of PBE. BiBit resulted in a huge number of small biclusters filled only with 1 (1,227 biclusters, row size = 13.5 and column size = 12.4).

For HOCCLUS2 biclusters, the size and density depended on the bicluster overlapping levels and α (cohesiveness). For all α, the level 1 biclusters were small (row size=13, column size = 10) with 100% density. As the level was increased, most biclusters became larger containing more zero proportions except for the cases of α = 0.8 and 0.9. The level 2 biclusters had 23 conditions and 19 genes on average for α = 0.4 - 0.7 and were most similar to those of PBE biclusters. However, their average density was rather lower (84.4% - 86.3%) compared with that of PBE biclusters (98.1%). From level 3 (α = 0.4 - 0.7), the biclusters showed very low densities which were far from useful to predict regulatory modules (Table S3).

All methods found homogenous biclusters whose cell conditions were mostly ESC/iPSC vs. somatic cell conditions. PBE showed the best performance with respect to size and/or density (51 conditions and 126 genes with 97.6% density) compared with other methods. The largest ISA bicluster had 71 conditions and 154 genes with only 83.4% density when T_C_ = 2, T_G_ = 1, and the densest one had only 35 conditions and 37 genes with 95.6% density when T_C_ = 4, T_G_ = 2. QUBIC bicluster had 47 conditions and 109 genes with 98.2% density. FABIA generated big bicluster (44 conditions and 293 genes) but the density was relatively low (80.2%) and BiBit yielded small bicluster (23

**Figure S4.** ESC/iPSC biclusters searched by multiple biclustering methods. All biclustering methods detected biclusters containing homogeneous ESC/iPSC vs. somatic cell conditions. (a) PBE detected large and dense bicluster (51 conditions and 126 targets with 97.6% density). (b) QUBIC detected rather small but dense bicluster (47 conditions and 109 targets with 98.2% density) (c) ISA found large but noisy bicluster (71 conditions and 154 targets with 83.4% density). (d) The densest ISA biclusters had a relatively small size (35 conditions and 37 targets with 95.6% density). (e) BiBit detected a small bicluster with 100% density (23 conditions and 35 genes) (f) HOCCLUS2 found a dense bicluster but the size was quite small (26 conditions and 84 genes with 97.3% density) (g) FABIA detected a large but very noisy bicluster (44 conditions and 293 targets 80.2% density)

condition and 35 genes) with 100% density. The level 2 bicluster of HOCCLUS2 had only 26 conditions and 84 genes with 97.3% density (a= 0.4 - 0.9) (Figure S4).

**Table S3.** Test results for real data. For PBE, QUBIC, BiBit, FABIA, ISA and HOCCLUS2, the average size of row/column, density, and the number of biclusters were measured using up-regulated MIR profile of hsa-let-7c-5p.

| **PBE** | | | | | | | | | |
| --- | --- | --- | --- | --- | --- | --- | --- | --- | --- |
|  | | **Row** | | | **Column** | | **Density** | **N** | |
|  |  | 23.3 | | | 38.9 | | 98.1 | 17 | |
| **QUBIC** | | | | | | | | | |
| **Consistency** | | **Row** | | | **Column** | | **Density** | **N** | |
| **1.0** | | 17.2 | | | 41.9 | | 1.0 | 46 | |
| **0.98** | | 16.9 | | | 42.0 | | 0.9997 | 44 | |
| **0.95** | | 14.2 | | | 43.4 | | 0.9989 | 36 | |
| **0.92** | | 15.2 | | | 61.3 | | 0.9796 | 24 | |
| **BiBit (minimum row and column size = 10)** | | | | | | | | | |
|  | | **Row** | | | **Column** | | **Density** | **N** | |
|  |  | 13.5 | | | 12.4 | | 1.0 | 1227 | |
| **FABIA** | | | | | | | | | |
|  | | **Continuous input** | | | | **Binary input** | | | |
| **Sparseness loading** | | **Row** | **Column** | **Density** | **N** | **Row** | **Column** | **Density** | **N** |
| **0.01** | | 80.1 | 219.4 | 0.318 | 30 | 55.5 | 226.3 | 0.358 | 22 |
| **0.05** | | 64.1 | 195.5 | 0.330 | 26 | 25.8 | 291.1 | 0.548 | 27 |
| **0.1** | | 25.9 | 189.6 | 0.355 | 26 | 15 | 285.2 | 0.653 | 25 |
| **0.15** | | 28.5 | 198.3 | 0.359 | 28 | 10.9 | 265.6 | 0.714 | 23 |
| **0.2** | | 25.8 | 198.7 | 0.368 | 25 | 12.2 | 268.1 | 0.723 | 15 |
| **0.25** | | 19.1 | 201.2 | 0.388 | 27 | 14.4 | 272 | 0.701 | 11 |
| **0.3** | | 28.2 | 195.8 | 0.361 | 23 | 11.9 | 266.8 | 0.719 | 10 |
| **ISA** | | | | | | | | | |
|  |  | **Continuous input** | | | | **Binary input** | | | |
| **T_G_** | **T_C_** | **Row** | **Column** | **Density** | **N** | **Row** | **Column** | **Density** | **N** |
| **1.0** | **1.0** | 174.0 | 119.3 | 0.500 | 4 | 192.7 | 116.2 | 0.464 | 6 |
|  | **1.5** | 176.7 | 60.7 | 0.526 | 7 | 191.8 | 70.2 | 0.498 | 9 |
|  | **2.0** | 196.5 | 27.7 | 0.493 | 15 | 200.8 | 39.5 | 0.534 | 13 |
|  | **2.5** | 202.8 | 11.4 | 0.546 | 22 | 216.9 | 18.9 | 0.532 | 22 |
|  | **3.0** | 189.5 | 5.6 | 0.672 | 28 | 221.0 | 10.2 | 0.582 | 18 |
| **1.5** | **1.0** | 106.4 | 118.6 | 0.486 | 7 | 95.0 | 118.0 | 0.486 | 9 |
|  | **1.5** | 106.7 | 60.1 | 0.530 | 12 | 94.8 | 66.8 | 0.511 | 9 |
|  | **2.0** | 100.1 | 28.3 | 0.582 | 15 | 113.2 | 39.5 | 0.560 | 11 |
|  | **2.5** | 101.6 | 12.4 | 0.623 | 22 | 127.7 | 21.2 | 0.591 | 24 |
|  | **3.0** | 105.2 | 6.2 | 0.707 | 23 | 133.6 | 11.1 | 0.647 | 16 |
| **2.0** | **1.0** | 58.1 | 112.0 | 0.482 | 11 | 59.2 | 113.8 | 0.509 | 12 |
|  | **1.5** | 52.5 | 58.4 | 0.554 | 17 | 58.3 | 69.5 | 0.533 | 13 |
|  | **2.0** | 52.3 | 27.0 | 0.621 | 21 | 72.4 | 43.2 | 0.605 | 11 |
|  | **2.5** | 54.3 | 12.3 | 0.641 | 28 | 66.6 | 20.9 | 0.569 | 29 |
|  | **3.0** | 59.2 | 8.1 | 0.744 | 18 | 74.1 | 13.0 | 0.676 | 18 |
| **2.5** | **1.0** | 25.8 | 110.8 | 0.529 | 30 | 28.6 | 109.0 | 0.529 | 24 |
|  | **1.5** | 25.8 | 58.3 | 0.632 | 28 | 32.7 | 71.3 | 0.581 | 23 |
|  | **2.0** | 32.0 | 28.3 | 0.703 | 22 | 33.0 | 42.1 | 0.599 | 29 |
|  | **2.5** | 34.9 | 14.3 | 0.719 | 21 | 38.6 | 22.0 | 0.635 | 30 |
|  | **3.0** | 37.2 | 8.7 | 0.770 | 13 | 45.7 | 10.1 | 0.694 | 44 |
| **3.0** | **1.0** | 14.7 | 105.2 | 0.638 | 37 | 15.0 | 120.6 | 0.619 | 41 |
|  | **1.5** | 17.1 | 52.7 | 0.701 | 33 | 16.1 | 72.5 | 0.645 | 45 |
|  | **2.0** | 18.3 | 27.5 | 0.744 | 32 | 18.4 | 42.7 | 0.658 | 42 |
|  | **2.5** | 23.0 | 14.9 | 0.804 | 19 | 21.2 | 25.2 | 0.654 | 46 |
|  | **3.0** | 26.1 | 9.2 | 0.806 | 9 | 26.9 | 12.3 | 0.669 | 43 |
| **HOCCLUS2** | | | | | | | | | |
| **Level** | **Beta** | **Row** | | **Column** | | **Density** | | **N** | |
| **1** | 0.4 | 13 | | 10 | | 1.0 | | 60 | |
|  | 0.5 | 13 | | 10 | | 1.0 | | 60 | |
|  | 0.6 | 13 | | 10 | | 1.0 | | 60 | |
|  | 0.7 | 13 | | 10 | | 1.0 | | 60 | |
|  | 0.8 | 13 | | 10 | | 1.0 | | 60 | |
|  | 0.9 | 13 | | 10 | | 1.0 | | 60 | |
| **2** | 0.4 | 23.5 | | 19 | | 0.844 | | 30 | |
|  | 0.5 | 23.5 | | 19 | | 0.844 | | 30 | |
|  | 0.6 | 23 | | 19 | | 0.855 | | 31 | |
|  | 0.7 | 23 | | 18.5 | | 0.863 | | 32 | |
|  | 0.8 | 19 | | 16.5 | | 0.986 | | 40 | |
|  | 0.9 | 12 | | 11 | | 1.0 | | 53 | |
| **3** | 0.4 | 45 | | 38 | | 0.687 | | 15 | |
|  | 0.5 | 45 | | 38 | | 0.687 | | 15 | |
|  | 0.6 | 41.5 | | 33 | | 0.742 | | 18 | |
|  | 0.7 | 24 | | 19 | | 0.805 | | 25 | |
|  | 0.8 | 18 | | 17 | | 1.0 | | 35 | |
|  | 0.9 | 12 | | 10.5 | | 1.0 | | 52 | |
| **4** | 0.4 | 80 | | 63 | | 0.578 | | 8 | |
|  | 0.5 | 71 | | 58 | | 0.575 | | 9 | |
|  | 0.6 | 45 | | 40 | | 0.682 | | 13 | |
|  | 0.7 | 23 | | 19 | | 0.797 | | 22 | |
|  | 0.8 | 18 | | 17 | | 1.0 | | 34 | |
|  | 0.9 | 12 | | 10 | | 1.0 | | 51 | |

**S5. Example: hsa-let-7c and pluripotency**

Let-7 is known to play an essential role in differentiation of embryonic stem cells (ESCs). Sustained expression of let-7 inhibits the reprogramming, and its inhibition promotes the human induced pluripotent stem cell (iPSC) reprogramming(28). PBE algorithm was applied to the let-7c MIR profile, and we found a stem cell specific bicluster comprising 126 target genes and 51 FC conditions (Figure S4a and Figure S6). This bicluster was quite homogeneous in that 50 of the 51 FC conditions were ESC/iPSC (test) *vs*. somatic cell (control) conditions suggesting many of the 126 genes are specifically regulated by let-7c or its family microRNAs in stem cells. Indeed, these targets included 49 genes that were reported to have a specific role in ESC (e.g., self-renewal) or upregulated in ESC (Table S4). Among them, 21 genes (ACVR2B, ARID3B, (29)CCND2, CCNF, CDC25A, DIAPH2, E2F5, HMGA1, IGF2BP1, IGF2BP3, LIN28A, LIN28B, MAPK6, MYCN, PAK1, POU2F1, SERPINB9, SLC5A6, STRBP, USP44 and VAV3) were validated targets of let-7(30-45). In particular, MYCN is regulated by let-7 under ESC condition(46), and LIN28B is also fine-tuned by let-7 in hESC(29). PLAGL2 which promotes self-renewal in neural stem cell and glioma was also a known target of let-7(47). This illustrates the capability of bicluster analysis to identify a specific regulatory module.

**Table S4.** Let-7c bicluster targets regulating pluripotency or up-regulated in ES/iPS cells. Genes reported to be regulated by let-7 is marked in bold.

| Gene symbol | Description | Ref. |
| --- | --- | --- |
| **ACVR2B** | Activin A binds to ActRIIA or ActRIIB and recruits ALK4. ALK4 interacts with SMAD2/3 and activates FGF2 pathways that stimulates self-renewal in human iPS cells by activating target genes including Nanog. | (48) |
| ACTA1 | Overexpressed in pooled human ES cells compared to huURNA (universal human reference RNA) | (49) |
| AMT | Overexpressed in pooled human ES cells compared to huURNA | (49) |
| ANKRD46 | Ankyrin repeat domain 46. It shows lower CpG methylation and higher gene expression level in pluripotent stem cell compared to somatic cell. | (50) |
| **ARID3B** | ARID3B complex regulates the expression of stemess genes and upregulates the let-7 target genes. Multiple steps in biogenesis of ARID3B-ARID3A complex are regulated by let-7. | (51) |
| B3GNT7 | The gene expression level of B3GNT7 was 5.84 and 3.16-fold higher in BG02 and BG01 human ES cell line, respectively, compared with the huURNA. | (52) |
| C6orf211 | Overexpressed in pooled human ES cells compared to huURNA | (49) |
| **CCND2** | CCND2 is a common target of OCT4, SOX2 and NANOG and its overexpression enhances the regenerative potency of hIPSC-derived cardiomyocytes. | (53,54) |
| **CCNF** | CCNF (Cyclin F) plays a role in cell cycle event and is essential for embryonic development. | (55) |
| **CDC25A** | NANOG regulates S-phase entry in human ES cells through direct binding of two cell cycle genes CDK6 and CDC25A | (56) |
| CDH1 | CDH1 regulates open chromatin and pluripotency of embryonic stem cell. | (57) |
| CDYL | CDYL is involved in histone modification. It inhibits the neuronal differentiation of iPS cells. | (58,59) |
| CTPS2 | Overexpressed in pooled human ES cells compared to huURNA | (49) |
| **DIAPH2** | DIAPH2 is involved in actin cytoskeleton pathway and specifically expressed in ES cells. | (33,60) |
| **E2F5** | E2F4, E2F5 and E2F6 may control E2F target genes during the DNA damage response in human ES cells | (61) |
| FZD3 | Overexpressed in pooled human ES cells compared to huURNA | (49) |
| GALNT13 | Overexpressed in pooled human ES cells compared to huURNA | (49) |
| GYG2 | Overexpressed in pooled human ES cells compared to huURNA | (49) |
| HIC2 | Overexpressed in pooled human ES cells compared to huURNA | (49) |
| **HMGA1** | HMGA1 is a transcription factor highly expressed in ES cells. | (62) |
| HOMER1 | Overexpressed in pooled human ES cells compared to huURNA | (49) |
| **IGF2BP1** | IGF2BP1 is highly expressed in ES cells and have important role in human pluripotent stem cell survival. | (63) |
| **IGF2BP3** | IGFBP3 is highly expressed in ES cells compared to differentiated cells. | (64) |
| IGSF1 | Overexpressed in pooled human ES cells compared to huURNA | (49) |
| KIAA1274 | Overexpressed in pooled human ES cells compared to huURNA | (49) |
| **LIN28A** | LIN28A regulates mouse iPSC metabolism by let-7-dependent and -independent manner. It is also involved in nucleologenesis during early embryonic development. | (65,66) |
| **LIN28B** | LIN28B have equivalent function with LIN28A | (66) |
| **MAPK6** | Disruption of PI3K/Akt, MAPK/ERK and NFkB signaling pathway results in loss of pluripotency and/or loss of viability. Expression level of MAPK6 was downregulated during the differentiation process. | (67) |
| MCM5 | MCM5 is involved in DNA replication and up-regulated during the initiation phase of reprogramming. | (68) |
| MED28 | A mediator subunit, MED28, is required for the acquisition and maintenance of pluripotency during reprogramming | (69) |
| **MYCN** | MYCN maintains embryonic stem cell pluripotency and self-renewal and regulated by let-7. | (46,70) |
| NAP1L1 | NAL1L1 regulates the proliferation of murine iPS cells | (71) |
| **PAK1** | PAK1 is involved in actin cytoskeleton pathway and regulates self-renewal activity | (33,72) |
| PLA2G3 | Overexpressed in pooled human ES cells compared to huURNA | (49) |
| **PLAGL2** | PLAGL2 promotes self-renewal by regulating Wnt signaling in neural stem cells and glioma | (73) |
| **POU2F1** | Overexpressed in pooled human ES cells compared to huURNA | (49) |
| PPP1R16B | PPP1R16B is hypo-methylated and highly expressed in iPS and ES cells | (74) |
| RFWD3 | Overexpressed in pooled human ES cells compared to huURNA | (49) |
| **SERPINB9** | Overexpressed in pooled human ES cells compared to huURNA | (49,75) |
| SLC16A9 | SLC16A9 is a downstream target of OCT4 and upregulated in ES cells. | (76) |
| **SLC5A6** | The gene expression level of SLC5A6was 3.06 and 3.46-fold higher in BG02 and BG01 hES cell line, respectively, compared with the huRNA (universal human RNA) | (52) |
| SMARCAD1 | SMARCAD1 regulates naïve pluripotency by interacting with histone citrullination. | (77) |
| SMARCC | SMARCC1 is involved in chromatin remodeling and highly induced in iPS cells | (78) |
| **STRBP** | Overexpressed in pooled human ES cells compared to huURNA | (49) |
| TAF5 | TAFs are highly expressed in ES and iPS cells and regulates pluripotency. | (79) |
| TARBP2 | Overexpressed in pooled human ES cells compared to huURNA | (49) |
| TIA1 | Overexpressed in pooled human ES cells compared to huURNA | (49) |
| THAP9 | Overexpressed in pooled human ES cells compared to huURNA | (49) |
| **USP44** | USP44 is highly expressed in ES and IPS cells and it regulates histone H2B ubiquitylation patterns for appropriate ESC differentiation. | (80,81) |
| **VAV3** | Overexpressed in pooled human ES cells compared to huURNA | (49) |

**Table S7.** microRNA expression patterns in cancers reported from the literature

| microRNA | Cancer | Direction | Reference |
| --- | --- | --- | --- |
| hsa-miR-1-3p | Breast cancer | Down-regulation | (82) |
| hsa-miR-21-5p | Breast cancer | Up-regulation | (83) |
| hsa-miR-29a-3p | AML | Down-regulation | (84,85) |
|  | Breast cancer | Down-regulation | (86) |
|  | DLBCL | Down-regulation | (87) |
|  | Glioblastoma/glioma | Down-regulation | (88) |
| hsa-miR-29b-3p | AML | Down-regulation | (89) |
|  | Breast cancer | Down-regulation | (90) |
|  | DLBCL | Down-regulation | (87) |
|  | Glioblastoma/glioma | Down-regulation | (91) |
| hsa-miR-29c-3p | Breast cancer | Down-regulation | (92) |
|  | DLBCL | Down-regulation | (87) |
|  | Glioblastoma/glioma | Down-regulation | (91) |
| hsa-miR-34a-5p | Breast cancer | Down-regulation | (93) |
|  | DLBCL | Down-regulation | (94) |
| hsa-miR-125a-5p | AML | Up-regulation | (95) |
| hsa-miR-145-5p | AML | Down-regulation | (96) |
|  | Breast cancer | Down-regulation | (97) |
|  | DLBCL | Down-regulation | (98) |
| hsa-miR-155-5p | Breast cancer | Up-regulation | (99) |
| hsa-miR-221-3p | Breast cancer | Up-regulation | (100) |

**Figure S5. microRNA targets in PI3K/Akt pathway (DLBCL).** (a) MicroRNA targets predicted from DLBCL biclusters in PI3K/Akt pathway are highlighted by red borders. For each target molecule, corresponding microRNAs and target gene symbols are represented. (b, c) Overall survival analysis for the 116 DLBCL patients (GSE40239) of high (red) and low (blue) (b) miR-34a and (c) miR-145 expression levels. The patients were divided into two groups based on their best splits (both at bottom 20% values).

**Table S10.** Multivariate Cox regression analysis of microRNAs in the DLBCL dataset

| **Variable** | **Hazard ratio** | **95% CI. *** | **p-value** |
| --- | --- | --- | --- |
| ***miR-29a*** |  |  |  |
| miR-29a | 0.903 | 0.662-1.231 | 5.18.E-01 |
| IPI * | 1.720 | 1.231-2.404 | 1.48.E-03 |
| Gender | 2.498 | 1.043-5.982 | 3.98.E-02 |
| ***miR-29b*** |  |  |  |
| miR-29b | 0.912 | 0.664-1.252 | 5.68.E-01 |
| IPI | 1.751 | 1.27-2.412 | 6.20.E-04 |
| Gender | 2.558 | 1.063-6.154 | 3.61.E-02 |
| ***miR-29c*** |  |  |  |
| miR-29c | 0.833 | 0.581-1.193 | 3.19.E-01 |
| IPI | 1.721 | 1.242-2.386 | 1.12.E-03 |
| Gender | 2.609 | 1.084-6.277 | 3.23.E-02 |
| ***miR-34a*** |  |  |  |
| miR-34a | 0.691 | 0.508-0.94 | **1.85.E-02** |
| IPI | 1.687 | 1.225-2.322 | 1.35.E-03 |
| Gender | 2.983 | 1.171-7.6 | 2.20.E-02 |
| ***miR-145*** |  |  |  |
| miR-145 | 0.593 | 0.415-0.848 | **4.13.E-03** |
| IPI | 1.787 | 1.312-2.434 | 2.28.E-04 |
| Gender | 3.075 | 1.266-7.466 | 1.31.E-02 |

*CI=Confidence Interval, IPI=International prognostic index

**Table S11. Multivariate Cox regression analysis of microRNAs in the breast cancer dataset**

|  | **Hazard ratio** | **95% CI. *** | **p-value** |
| --- | --- | --- | --- |
| ***miR-1*** |  |  |  |
| miR-1 | 1.034 | 0.848-1.261 | 7.43.E-01 |
| Age | 1.036 | 1.011-1.061 | 4.22.E-03 |
| Tumor size | 1.206 | 1.018-1.43 | 3.08.E-02 |
| Lymph nodes involved | 1.194 | 1.125-1.268 | 6.13.E-09 |
| ER * | 0.666 | 0.396-1.12 | 1.25.E-01 |
| Grade | 1.603 | 1.099-2.339 | 1.43.E-02 |
| ***miR-29a*** |  |  |  |
| miR-29a | 0.745 | 0.609-0.911 | **4.22.E-03** |
| Age | 1.039 | 1.014-1.065 | 2.36.E-03 |
| Tumor size | 1.213 | 1.03-1.428 | 2.05.E-02 |
| Lymph nodes involved | 1.213 | 1.141-1.289 | 5.70.E-10 |
| ER | 0.605 | 0.356-1.027 | 6.28.E-02 |
| Grade | 1.477 | 1.012-2.156 | 4.29.E-02 |
| ***miR-29b*** |  |  |  |
| miR-29b | 0.717 | 0.565-0.911 | **6.42.E-03** |
| Age | 1.041 | 1.016-1.067 | 1.02.E-03 |
| Tumor size | 1.245 | 1.058-1.465 | 8.40.E-03 |
| Lymph nodes involved | 1.209 | 1.136-1.287 | 2.08.E-09 |
| ER | 0.713 | 0.424-1.2 | 2.03.E-01 |
| Grade | 1.712 | 1.176-2.49 | 4.96.E-03 |
| ***miR-29c*** |  |  |  |
| miR-29c | 0.715 | 0.57-0.897 | **3.80.E-03** |
| Age | 1.037 | 1.014-1.061 | 1.75.E-03 |
| Tumor size | 1.256 | 1.064-1.483 | 7.15.E-03 |
| Lymph nodes involved | 1.195 | 1.127-1.267 | 2.06.E-09 |
| ER | 0.796 | 0.467-1.356 | 4.01.E-01 |
| Grade | 1.469 | 1.012-2.13 | 4.29.E-02 |
| ***miR-34a*** |  |  |  |
| miR-34a | 1.023 | 0.795-1.316 | 8.62.E-01 |
| Age | 1.036 | 1.011-1.062 | 4.10.E-03 |
| Tumor size | 1.216 | 1.034-1.429 | 1.80.E-02 |
| Lymph nodes involved | 1.193 | 1.124-1.266 | 5.43.E-09 |
| ER | 0.669 | 0.398-1.124 | 1.29.E-01 |
| Grade | 1.573 | 1.083-2.286 | 1.75.E-02 |
| ***miR-145*** |  |  |  |
| miR-145 | 1.168 | 0.921-1.482 | 2.00.E-01 |
| Age | 1.038 | 1.013-1.063 | 2.92.E-03 |
| Tumor size | 1.224 | 1.042-1.437 | 1.37.E-02 |
| Lymph nodes involved | 1.195 | 1.126-1.267 | 2.90.E-09 |
| ER | 0.702 | 0.418-1.181 | 1.83.E-01 |
| Grade | 1.631 | 1.126-2.362 | 9.69.E-03 |

*CI=Confidence Interval, ER=Estrogen receptor

**S6. MicroRNA regulation of PI3K/Akt pathways in the literature**

The microRNAs detected in cancer biclusters were able to suppress PI3K/Akt pathway and metastasis in multiple cancer types. For example, up-regulated miR-29a inhibited the lung cancer proliferation by targeting NRAS which is a key downstream effector of PI3K/Akt pathway(101). Up-regulated MiR-29b suppressed the breast cancer metastasis by targeting VEGFA, PDGFC and ITGB1(102), and it also reduced angiogenesis of endometrial cancer by targeting VEGFA(103). MiR-34a inhibited gastric cancer growth, invasion, and metastasis by targeting two signal transducers of the pathway, PDGFR and MET(104). Mir-1 also acted as a tumor suppressor in gastric cancer by targeting VEGFA and MET(105,106). Lastly, miR-145 inhibited PI3K/Akt pathway by targeting NRAS in melanoma(107). The same targets and microRNAs were detected in our bicluster results for breast cancer and DLBCL, suggesting these microRNAs are also able to suppress PI3K/Akt pathway and metastasis in these cancer types. Indeed, it was shown in vivo that mir-29b considerably inhibits breast cancer metastasis by suppressing tumor microenvironment related targets(102). Our biclustering result suggests collagen and other genes in PI3K/Akt pathway are also targets of mir-29 in breast cancer and DLBCL.


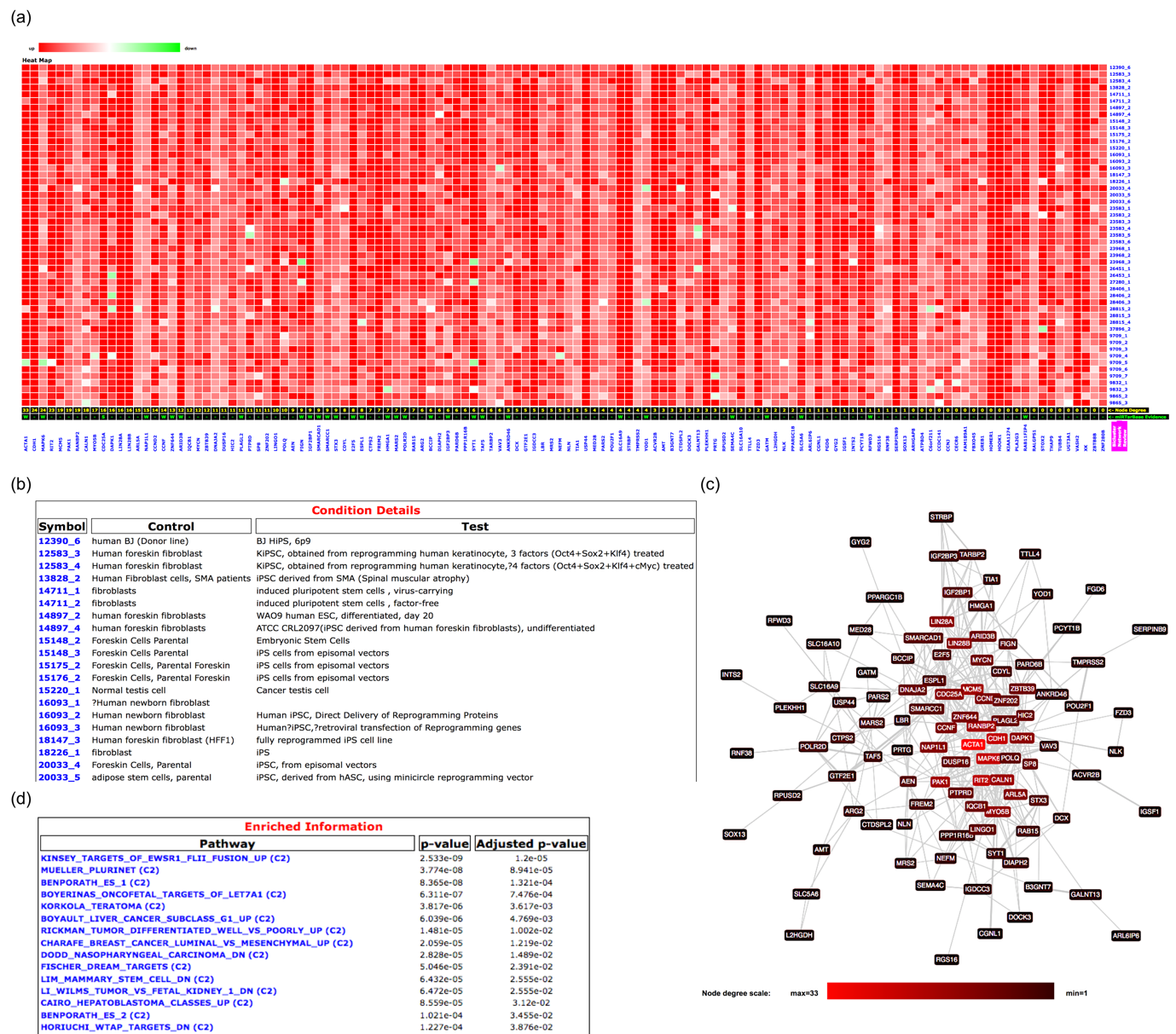


**Figure S6.** BiMIR database. (a) Heatmap of hsa-let-7c-5p bicluster up-regulated under embryonic stem cell/iPS cell/somatic cell conditions. Row and column represent the symbols of experimental conditions and target gene symbols, respectively. For each target gene, the user can check the node-degree for target PPI network and whether it is experimentally validated. (b) Detailed condition information (test and control group info.) is represented. Wordcloud for conditions is also provided. (c) PPI network for bicluster targets are visualized. The nodes with bright red color are connected with many other targets.

**REFERENCES**

1. Garcia, D.M., Baek, D., Shin, C., Bell, G.W., Grimson, A. and Bartel, D.P. (2011) Weak seed-pairing stability and high target-site abundance decrease the proficiency of lsy-6 and other microRNAs. *Nat Struct Mol Biol*, **18**, 1139-1146.

2. Kertesz, M., Iovino, N., Unnerstall, U., Gaul, U. and Segal, E. (2007) The role of site accessibility in microRNA target recognition. *Nature Genetics*, **39**, 1278-1284.

3. Wang, X.W. (2016) Improving microRNA target prediction by modeling with unambiguously identified microRNA-target pairs from CLIP-ligation studies. *Bioinformatics (Oxford, England)*, **32**, 1316-1322.

4. Betel, D., Koppal, A., Agius, P., Sander, C. and Leslie, C. (2010) Comprehensive modeling of microRNA targets predicts functional non-conserved and non-canonical sites. *Genome Biol*, **11**, R90.

5. Kozomara, A. and Griffiths-Jones, S. (2014) miRBase: annotating high confidence microRNAs using deep sequencing data. *Nucleic acids research*, **42**, D68-73.

6. Maragkakis, M., Reczko, M., Simossis, V.A., Alexiou, P., Papadopoulos, G.L., Dalamagas, T., Giannopoulos, G., Goumas, G., Koukis, E., Kourtis, K. *et al.* (2009) DIANA-microT web server: elucidating microRNA functions through target prediction. *Nucleic acids research*, **37**, W273-W276.

7. Paraskevopoulou, M.D., Georgakilas, G., Kostoulas, N., Vlachos, I.S., Vergoulis, T., Reczko, M., Filippidis, C., Dalamagas, T. and Hatzigeorgiou, A.G. (2013) DIANA-microT web server v5.0: service integration into miRNA functional analysis workflows. *Nucleic acids research*, **41**, W169-W173.

8. Nielsen, C.B., Shomron, N., Sandberg, R., Hornstein, E., Kitzman, J. and Burge, C.B. (2007) Determinants of targeting by endogenous and exogenous microRNAs and siRNAs. *Rna*, **13**, 1894-1910.

9. Wang, Y.P. and Li, K.B. (2009) Correlation of expression profiles between microRNAs and mRNA targets using NCI-60 data. *Bmc Genomics*, **10**.

10. Ng, E.K., Tsang, W.P., Ng, S.S., Jin, H.C., Yu, J., Li, J.J., Rocken, C., Ebert, M.P., Kwok, T.T. and Sung, J.J. (2009) MicroRNA-143 targets DNA methyltransferases 3A in colorectal cancer. *Br J Cancer*, **101**, 699-706.

11. Santosa, F. and Symes, W.W. (1986) Linear Inversion of Band-Limited Reflection Seismograms. *Siam Journal on Scientific and Statistical Computing*, **7**, 1307-1330.

12. Tibshirani, R. (1996) Regression shrinkage and selection via the Lasso. *Journal of the Royal Statistical Society Series B-Methodological*, **58**, 267-288.

13. Sass, S., Pitea, A., Unger, K., Hess, J., Mueller, N.S. and Theis, F.J. (2015) MicroRNA-Target Network Inference and Local Network Enrichment Analysis Identify Two microRNA Clusters with Distinct Functions in Head and Neck Squamous Cell Carcinoma. *Int J Mol Sci*, **16**, 30204-30222.

14. Huang, J.C., Babak, T., Corson, T.W., Chua, G., Khan, S., Gallie, B.L., Hughes, T.R., Blencowe, B.J., Frey, B.J. and Morris, Q.D. (2007) Using expression profiling data to identify human microRNA targets. *Nature Methods*, **4**, 1045-1049.

15. Koo, J., Zhang, J.Y. and Chaterji, S. (2018) Tiresias: Context-sensitive Approach to Decipher the Presence and Strength of MicroRNA Regulatory Interactions. *Theranostics*, **8**, 277-291.

16. Le, T.D., Liu, L., Tsykin, A., Goodall, G.J., Liu, B., Sun, B.Y. and Li, J. (2013) Inferring microRNA-mRNA causal regulatory relationships from expression data. *Bioinformatics*, **29**, 765-771.

17. Pio, G., Ceci, M., D'Elia, D., Loglisci, C. and Malerba, D. (2013) A novel biclustering algorithm for the discovery of meaningful biological correlations between microRNAs and their target genes. *BMC Bioinformatics*, **14 Suppl 7**, S8.

18. Yoon, S. and De Micheli, G. (2005) Prediction of regulatory modules comprising microRNAs and target genes. *Bioinformatics (Oxford, England)*, **21 Suppl 2**, ii93-100.

19. Bryan, K., Terrile, M., Bray, I.M., Domingo-Fernandez, R., Watters, K.M., Koster, J., Versteeg, R. and Stallings, R.L. (2014) Discovery and visualization of miRNA-mRNA functional modules within integrated data using bicluster analysis. *Nucleic Acids Res*, **42**, e17.

20. Joung, J.G., Hwang, K.B., Nam, J.W., Kim, S.J. and Zhang, B.T. (2007) Discovery of microRNA-mRNA modules via population-based probabilistic learning. *Bioinformatics (Oxford, England)*, **23**, 1141-1147.

21. Liu, B., Liu, L., Tsykin, A., Goodall, G.J., Green, J.E., Zhu, M., Kim, C.H. and Li, J. (2010) Identifying functional miRNA-mRNA regulatory modules with correspondence latent dirichlet allocation. *Bioinformatics (Oxford, England)*, **26**, 3105-3111.

22. Jayaswal, V., Lutherborrow, M., Ma, D.D. and Yang, Y.H. (2011) Identification of microRNA-mRNA modules using microarray data. *BMC genomics*, **12**, 138.

23. Bergmann, S., Ihmels, J. and Barkai, N. (2003) Iterative signature algorithm for the analysis of large-scale gene expression data. *Physical Review E*, **67**.

24. Li, G.J., Ma, Q., Tang, H.B., Paterson, A.H. and Xu, Y. (2009) QUBIC: a qualitative biclustering algorithm for analyses of gene expression data. *Nucleic Acids Res*, **37**.

25. Hochreiter, S., Bodenhofer, U., Heusel, M., Mayr, A., Mitterecker, A., Kasim, A., Khamiakova, T., Van Sanden, S., Lin, D., Talloen, W. *et al.* (2010) FABIA: factor analysis for bicluster acquisition. *Bioinformatics (Oxford, England)*, **26**, 1520-1527.

26. Rodriguez-Baena, D.S., Perez-Pulido, A.J. and Aguilar-Ruiz, J.S. (2011) A biclustering algorithm for extracting bit-patterns from binary datasets. *Bioinformatics (Oxford, England)*, **27**, 2738-2745.

27. Gonzalez-Dominguez, J. and Exposit, R.R. (2018) ParBiBit: Parallel tool for binary biclustering on modern distributed-memory systems. *Plos One*, **13**.

28. Worringer, K.A., Rand, T.A., Hayashi, Y., Sami, S., Takahashi, K., Tanabe, K., Narita, M., Srivastava, D. and Yamanaka, S. (2014) The let-7/LIN-41 pathway regulates reprogramming to human induced pluripotent stem cells by controlling expression of prodifferentiation genes. *Cell Stem Cell*, **14**, 40-52.

29. Rahkonen, N., Stubb, A., Malonzo, M., Edelman, S., Emani, M.R., Narva, E., Lahdesmaki, H., Ruohola-Baker, H., Lahesmaa, R. and Lund, R. (2016) Mature Let-7 miRNAs fine tune expression of LIN28B in pluripotent human embryonic stem cells. *Stem Cell Res*, **17**, 498-503.

30. Zipeto, M.A., Court, A.C., Sadarangani, A., Delos Santos, N.P., Balaian, L., Chun, H.J., Pineda, G., Morris, S.R., Mason, C.N., Geron, I. *et al.* (2016) ADAR1 Activation Drives Leukemia Stem Cell Self-Renewal by Impairing Let-7 Biogenesis. *Cell Stem Cell*, **19**, 177-191.

31. Liao, T.T. and Yang, M.H. (2013) Downregulation of Let-7i to promote stem-like properties of head and neck cancer cells through activating ARID3B-Oct4 axis. *Journal of Clinical Oncology*, **31**.

32. Johnson, C.D., Esquela-Kerscher, A., Stefani, G., Byrom, M., Kelnar, K., Ovcharenko, D., Wilson, M., Wang, X., Shelton, J., Shingara, J. *et al.* (2007) The let-7 microRNA represses cell proliferation pathways in human cells. *Cancer Res*, **67**, 7713-7722.

33. Hu, X., Guo, J., Zheng, L., Li, C., Zheng, T.M., Tanyi, J.L., Liang, S., Benedetto, C., Mitidieri, M., Katsaros, D. *et al.* (2013) The heterochronic microRNA let-7 inhibits cell motility by regulating the genes in the actin cytoskeleton pathway in breast cancer. *Mol Cancer Res*, **11**, 240-250.

34. Chafin, C.B., Regna, N.L., Caudell, D.L. and Reilly, C.M. (2014) MicroRNA-let-7a promotes E2F-mediated cell proliferation and NFkappaB activation in vitro. *Cell Mol Immunol*, **11**, 79-83.

35. Liu, K., Zhang, C., Li, T., Ding, Y., Tu, T., Zhou, F., Qi, W., Chen, H. and Sun, X. (2015) Let-7a inhibits growth and migration of breast cancer cells by targeting HMGA1. *Int J Oncol*, **46**, 2526-2534.

36. Boyerinas, B., Park, S.M., Shomron, N., Hedegaard, M.M., Vinther, J., Andersen, J.S., Feig, C., Xu, J., Burge, C.B. and Peter, M.E. (2008) Identification of let-7-regulated oncofetal genes. *Cancer Res*, **68**, 2587-2591.

37. Rybak, A., Fuchs, H., Smirnova, L., Brandt, C., Pohl, E.E., Nitsch, R. and Wulczyn, F.G. (2008) A feedback loop comprising lin-28 and let-7 controls pre-let-7 maturation during neural stem-cell commitment. *Nat Cell Biol*, **10**, 987-993.

38. Elkhadragy, L., Chen, M., Miller, K., Yang, M.H. and Long, W. (2017) A regulatory BMI1/let-7i/ERK3 pathway controls the motility of head and neck cancer cells. *Mol Oncol*, **11**, 194-207.

39. Powers, J.T., Tsanov, K.M., Pearson, D.S., Roels, F., Spina, C.S., Ebright, R., Seligson, M., de Soysa, Y., Cahan, P., Theissen, J. *et al.* (2016) Multiple mechanisms disrupt the let-7 microRNA family in neuroblastoma. *Nature*, **535**, 246-251.

40. Chen, K.C., Hsieh, I.C., Hsi, E., Wang, Y.S., Dai, C.Y., Chou, W.W. and Juo, S.H.H. (2011) Negative feedback regulation between microRNA let-7g and the oxLDL receptor LOX-1. *Journal of Cell Science*, **124**, 4115-4124.

41. Russ, A.C., Sander, S., Luck, S.C., Lang, K.M., Bauer, M., Rucker, F.G., Kestler, H.A., Schlenk, R.F., Dohner, H., Holzmann, K. *et al.* (2011) Integrative nucleophosmin mutation-associated microRNA and gene expression pattern analysis identifies novel microRNA - target gene interactions in acute myeloid leukemia. *Haematologica*, **96**, 1783-1791.

42. Lin, L.T., Chang, C.Y., Chang, C.H., Wang, H.E., Chiou, S.H., Liu, R.S., Lee, T.W. and Lee, Y.J. (2016) Involvement of let-7 microRNA for the therapeutic effects of Rhenium-188-embedded liposomal nanoparticles on orthotopic human head and neck cancer model. *Oncotarget*, **7**, 65782-65796.

43. Vaz, C., Ahmad, H.M., Sharma, P., Gupta, R., Kumar, L., Kulshreshtha, R. and Bhattacharya, A. (2010) Analysis of microRNA transcriptome by deep sequencing of small RNA libraries of peripheral blood. *BMC genomics*, **11**, 288.

44. Spolverini, A., Fuchs, G., Bublik, D.R. and Oren, M. (2017) let-7b and let-7c microRNAs promote histone H2B ubiquitylation and inhibit cell migration by targeting multiple components of the H2B deubiquitylation machinery. *Oncogene*, **36**, 5819-5828.

45. Cheong, W.A. (2013), University of Hong Kong, Hong Kong.

46. Melton, C., Judson, R.L. and Blelloch, R. (2010) Opposing microRNA families regulate self-renewal in mouse embryonic stem cells (vol 463, pg 621, 2010). *Nature*, **464**, 126-126.

47. Patterson, M., Gaeta, X., Loo, K., Edwards, M., Smale, S., Cinkornpumin, J., Xie, Y., Listgarten, J., Azghadi, S., Douglass, S.M. *et al.* (2014) let-7 miRNAs Can Act through Notch to Regulate Human Gliogenesis. *Stem Cell Reports*, **3**, 758-773.

48. Diecke, S., Quiroga-Negreira, A., Redmer, T. and Besser, D. (2008) FGF2 signaling in mouse embryonic fibroblasts is crucial for self-renewal of embryonic stem cells. *Cells Tissues Organs*, **188**, 52-61.

49. Bhattacharya, B., Cai, J., Luo, Y., Miura, T., Mejido, J., Brimble, S.N., Zeng, X., Schulz, T.C., Rao, M.S. and Puri, R.K. (2005) Comparison of the gene expression profile of undifferentiated human embryonic stem cell lines and differentiating embryoid bodies. *BMC Dev Biol*, **5**, 22.

50. Lenz, M., Goetzke, R., Schenk, A., Schubert, C., Veeck, J., Hemeda, H., Koschmieder, S., Zenke, M., Schuppert, A. and Wagner, W. (2015) Epigenetic Biomarker to Support Classification into Pluripotent and Non-Pluripotent Cells. *Scientific Reports*, **5**.

51. Liao, T.T., Hsu, W.H., Ho, C.H., Hwang, W.L., Lan, H.Y., Lo, T., Chang, C.C., Tai, S.K. and Yang, M.H. (2016) let-7 Modulates Chromatin Configuration and Target Gene Repression through Regulation of the ARID3B Complex. *Cell Reports*, **14**, 520-533.

52. Zeng, X.M., Miura, T., Luo, Y.Q., Bhattacharya, B., Condie, B., Chen, J., Ginis, I., Lyons, I., Mejido, J., Puri, R.K. *et al.* (2004) Properties of pluripotent human embryonic stem cells BG01 and BG02. *Stem Cells*, **22**, 292-312.

53. Zhu, W., Zhao, M., Mattapally, S., Chen, S. and Zhang, J. (2018) CCND2 Overexpression Enhances the Regenerative Potency of Human Induced Pluripotent Stem Cell-Derived Cardiomyocytes: Remuscularization of Injured Ventricle. *Circ Res*, **122**, 88-96.

54. Li, L.J., Chen, Z.B., Zhang, L.C., Liu, G.Y., Hua, J.L., Jia, L.H. and Liao, M.Z. (2016) Genome-wide targets identification of "core'' pluripotency transcription factors with integrated features in human embryonic stem cells. *Molecular Biosystems*, **12**, 1324-1332.

55. Tetzlaff, M.T., Bai, C., Finegold, M., Wilson, J., Harper, J.W., Mahon, K.A. and Elledge, S.J. (2004) Cyclin F disruption compromises placental development and affects normal cell cycle execution. *Mol Cell Biol*, **24**, 2487-2498.

56. Zhang, X., Neganova, I., Przyborski, S., Yang, C.B., Cooke, M., Atkinson, S.P., Anyfantis, G., Fenyk, S., Keith, W.N., Hoare, S.F. *et al.* (2009) A role for NANOG in G1 to S transition in human embryonic stem cells through direct binding of CDK6 and CDC25A. *Journal of Cell Biology*, **184**, 67-82.

57. Gaspar-Maia, A., Alajem, A., Polesso, F., Sridharan, R., Mason, M.J., Heidersbach, A., Ramalho-Santos, J., McManus, M.T., Plath, K., Meshorer, E. *et al.* (2009) Chd1 regulates open chromatin and pluripotency of embryonic stem cells. *Nature*, **460**, 863-868.

58. Wan, L., Hu, X.J., Yan, S.X., Chen, F., Cai, B., Zhang, X.M., Wang, T., Yu, X.B., Xiang, A.P. and Li, W.Q. (2013) Generation and neuronal differentiation of induced pluripotent stem cells in Cdyl-/- mice. *Neuroreport*, **24**, 114-119.

59. Boland, M.J., Nazor, K.L. and Loring, J.F. (2014) Epigenetic Regulation of Pluripotency and Differentiation. *Circulation Research*, **115**, 311-324.

60. Kumar, S., Curran, J.E., Glahn, D.C. and Blangero, J. (2016) Utility of Lymphoblastoid Cell Lines for Induced Pluripotent Stem Cell Generation. *Stem Cells Int*, **2016**, 2349261.

61. Becker, K.A., Stein, J.L., Lian, J.B., van Wijnen, A.J. and Stein, G.S. (2007) Establishment of histone gene regulation and cell cycle checkpoint control in human embryonic stem cells. *J Cell Physiol*, **210**, 517-526.

62. Richards, M., Tan, S.P., Tan, J.H., Chan, W.K. and Bongso, A. (2004) The transcriptome profile of human embryonic stem cells as defined by SAGE. *Stem Cells*, **22**, 51-64.

63. Conway, A.E., Van Nostrand, E.L., Pratt, G.A., Aigner, S., Wilbert, M.L., Sundararaman, B., Freese, P., Lambert, N.J., Sathe, S., Liang, T.Y. *et al.* (2016) Enhanced CLIP Uncovers IMP Protein-RNA Targets in Human Pluripotent Stem Cells Important for Cell Adhesion and Survival. *Cell Reports*, **15**, 666-679.

64. Bell, J.L., Wachter, K., Muhleck, B., Pazaitis, N., Kohn, M., Lederer, M. and Huttelmaier, S. (2013) Insulin-like growth factor 2 mRNA-binding proteins (IGF2BPs): post-transcriptional drivers of cancer progression? *Cell Mol Life Sci*, **70**, 2657-2675.

65. Vogt, E.J., Meglicki, M., Hartung, K.I., Borsuk, E. and Behr, R. (2012) Importance of the pluripotency factor LIN28 in the mammalian nucleolus during early embryonic development. *Development*, **139**, 4514-4523.

66. Zhang, J., Ratanasirintrawoot, S., Chandrasekaran, S., Wu, Z., Ficarro, S.B., Yu, C., Ross, C.A., Cacchiarelli, D., Xia, Q., Seligson, M. *et al.* (2016) LIN28 Regulates Stem Cell Metabolism and Conversion to Primed Pluripotency. *Cell Stem Cell*, **19**, 66-80.

67. Armstrong, L., Hughes, O., Yung, S., Hyslop, L., Stewart, R., Wappler, I., Peters, H., Walter, T., Stojkovic, P., Evans, J. *et al.* (2006) The role of PI3K/AKT, MAPK/ERK and NFkappabeta signalling in the maintenance of human embryonic stem cell pluripotency and viability highlighted by transcriptional profiling and functional analysis. *Hum Mol Genet*, **15**, 1894-1913.

68. Gonzalez, F. and Huangfu, D. (2016) Mechanisms underlying the formation of induced pluripotent stem cells. *Wiley Interdiscip Rev Dev Biol*, **5**, 39-65.

69. Li, L., Walsh, R.M., Wagh, V., James, M.F., Beauchamp, R.L., Chang, Y.S., Gusella, J.F., Hochedlinger, K. and Ramesh, V. (2015) Mediator Subunit Med28 Is Essential for Mouse Peri-Implantation Development and Pluripotency. *Plos One*, **10**.

70. Varlakhanova, N.V., Cotterman, R.F., deVries, W.N., Morgan, J., Donahue, L.R., Murray, S., Knowles, B.B. and Knoepfler, P.S. (2010) myc maintains embryonic stem cell pluripotency and self-renewal. *Differentiation*, **80**, 9-19.

71. Yan, Y., Yin, P.P., Gong, H., Xue, Y.Y., Zhang, G.P., Fang, B., Chen, Z.D., Li, Y., Yang, C.J., Huang, Z.Y. *et al.* (2016) Nucleosome Assembly Protein 1-Like 1 (Nap1l1) Regulates the Proliferation of Murine Induced Pluripotent Stem Cells. *Cellular Physiology and Biochemistry*, **38**, 340-350.

72. Zhu, Y., Liu, H., Xu, L., An, H., Liu, W., Liu, Y., Lin, Z. and Xu, J. (2015) p21-activated kinase 1 determines stem-like phenotype and sunitinib resistance via NF-kappaB/IL-6 activation in renal cell carcinoma. *Cell Death Dis*, **6**, e1637.

73. Zheng, H.W., Ying, H.Q., Wiedemeyer, R., Yan, H.Y., Quayle, S.N., Ivanova, E.V., Paik, J.H., Zhang, H.L., Xiao, Y.H., Perry, S.R. *et al.* (2010) PLAGL2 Regulates Wnt Signaling to Impede Differentiation in Neural Stem Cells and Gliomas. *Cancer Cell*, **17**, 497-509.

74. Nishino, K., Toyoda, M., Yamazaki-Inoue, M., Makino, H., Fukawatase, Y., Chikazawa, E., Takahashi, Y., Miyagawa, Y., Okita, H., Kiyokawa, N. *et al.* (2010) Defining hypo-methylated regions of stem cell-specific promoters in human iPS cells derived from extra-embryonic amnions and lung fibroblasts. *PLoS One*, **5**, e13017.

75. Pripuzova, N.S., Getie-Kebtie, M., Grunseich, C., Sweeney, C., Malech, H. and Alterman, M.A. (2015) Development of a protein marker panel for characterization of human induced pluripotent stem cells (hiPSCs) using global quantitative proteome analysis. *Stem Cell Research*, **14**, 323-338.

76. Awe, J.P., Crespo, A.V., Li, Y., Kiledjian, M. and Byrne, J.A. (2013) BAY11 enhances OCT4 synthetic mRNA expression in adult human skin cells. *Stem Cell Res Ther*, **4**, 15.

77. Xiao, S., Lu, J., Sridhar, B., Cao, X., Yu, P., Zhao, T., Chen, C.C., McDee, D., Sloofman, L., Wang, Y. *et al.* (2017) SMARCAD1 Contributes to the Regulation of Naive Pluripotency by Interacting with Histone Citrullination. *Cell Rep*, **18**, 3117-3128.

78. Huang, X., Tian, C.H., Liu, M., Wang, Y.X., Tolmachev, A.V., Sharma, S., Yu, F., Fu, K., Zheng, J.L. and Ding, S.J. (2012) Quantitative Proteomic Analysis of Mouse Embryonic Fibroblasts and Induced Pluripotent Stem Cells Using O-16/O-18 Labeling. *Journal of Proteome Research*, **11**, 2091-2102.

79. Baumann, K. (2013) Stem cells: TFIID promotes pluripotency. *Nat Rev Mol Cell Biol*, **14**, 264.

80. Tropel, P., Jung, L., Andre, C., Ndandougou, A. and Viville, S. (2017) CpG Island Methylation Correlates with the Use of Alternative Promoters for USP44 Gene Expression in Human Pluripotent Stem Cells and Testes. *Stem Cells and Development*, **26**, 1100-1110.

81. Fuchs, G., Shema, E., Vesterman, R., Kotler, E., Wolchinsky, Z., Wilder, S., Golomb, L., Pribluda, A., Zhang, F., Haj-Yahya, M. *et al.* (2012) RNF20 and USP44 regulate stem cell differentiation by modulating H2B monoubiquitylation. *Mol Cell*, **46**, 662-673.

82. Liu, R.L., Li, J., Lai, Y.H., Liao, Y., Liu, R.M. and Qiu, W.S. (2015) Hsa-miR-1 suppresses breast cancer development by down-regulating K-ras and long non-coding RNA MALAT1. *International Journal of Biological Macromolecules*, **81**, 491-497.

83. Yan, L.X., Huang, X.F., Shao, Q., Huang, M.Y., Deng, L., Wu, Q.L., Zeng, Y.X. and Shao, J.Y. (2008) MicroRNA miR-21 overexpression in human breast cancer is associated with advanced clinical stage, lymph node metastasis and patient poor prognosis. *Rna-a Publication of the Rna Society*, **14**, 2348-2360.

84. Marcucci, G., Mrozek, K., Radmacher, M.D., Garzon, R. and Bloomfield, C.D. (2011) The prognostic and functional role of microRNAs in acute myeloid leukemia. *Blood*, **117**, 1121-1129.

85. Zhu, C., Wang, Y., Kuai, W., Sun, X., Chen, H. and Hong, Z. (2013) Prognostic value of miR-29a expression in pediatric acute myeloid leukemia. *Clin Biochem*, **46**, 49-53.

86. Wu, Z., Huang, X., Huang, X., Zou, Q. and Guo, Y. (2013) The inhibitory role of Mir-29 in growth of breast cancer cells. *J Exp Clin Cancer Res*, **32**, 98.

87. Zhang, X.W., Zhao, X.H., Fiskus, W., Lin, J.H., Lwin, T., Rao, R., Zhang, Y.Z., Chan, J.C., Fu, K., Marquez, V.E. *et al.* (2012) Coordinated Silencing of MYC-Mediated miR-29 by HDAC3 and EZH2 as a Therapeutic Target of Histone Modification in Aggressive B-Cell Lymphomas. *Cancer Cell*, **22**, 506-523.

88. Xi, Z., Wang, P., Xue, Y., Shang, C., Liu, X., Ma, J., Li, Z., Li, Z., Bao, M. and Liu, Y. (2017) Overexpression of miR-29a reduces the oncogenic properties of glioblastoma stem cells by downregulating Quaking gene isoform 6. *Oncotarget*, **8**, 24949-24963.

89. Garzon, R., Heaphy, C.E., Havelange, V., Fabbri, M., Volinia, S., Tsao, T., Zanesi, N., Kornblau, S.M., Marcucci, G., Calin, G.A. *et al.* (2009) MicroRNA 29b functions in acute myeloid leukemia. *Blood*, **114**, 5331-5341.

90. Drago-Ferrante, R., Pentimalli, F., Carlisi, D., De Blasio, A., Saliba, C., Baldacchino, S., Degaetano, J., Debono, J., Caruana-Dingli, G., Grech, G. *et al.* (2017) Suppressive role exerted by microRNA-29b-1-5p in triple negative breast cancer through SPIN1 regulation. *Oncotarget*, **8**, 28939-28958.

91. Catania, A., Maira, F., Skarmoutsou, E., D'Amico, F., Abounader, R. and Mazzarino, M.C. (2012) Insight into the role of microRNAs in brain tumors (Review). *International Journal of Oncology*, **40**, 605-624.

92. Li, W., Yi, J., Zheng, X.J., Liu, S.W., Fu, W.Q., Ren, L.W., Li, L., Hoon, D.S.B., Wang, J.H. and Du, G.H. (2018) miR-29c plays a suppressive role in breast cancer by targeting the TIMP3/STAT1/FOXO1 pathway. *Clinical Epigenetics*, **10**.

93. Li, L.S., Yuan, L.J., Luo, J.M., Gao, J., Guo, J.L. and Xie, X.M. (2013) MiR-34a inhibits proliferation and migration of breast cancer through down-regulation of Bcl-2 and SIRT1. *Clinical and Experimental Medicine*, **13**, 109-117.

94. Liu, Y.P., Hu, H., Xu, F. and Wen, J.J. (2017) [Relation of MiR-34a Expression in Diffuse Large B Cell Lymphoma with Clinical Prognosis]. *Zhongguo Shi Yan Xue Ye Xue Za Zhi*, **25**, 455-459.

95. Romero, P.V., Cialfi, S., Palermo, R., De Blasio, C., Checquolo, S., Bellavia, D., Chiaretti, S., Foa, R., Amadori, A., Gulino, A. *et al.* (2015) The deregulated expression of miR-125b in acute myeloid leukemia is dependent on the transcription factor C/EBP alpha. *Leukemia*, **29**, 2442-2445.

96. Caramuta, S., Lee, L., Ozata, D.M., Akcakaya, P., Georgii-Hemming, P., Xie, H., Amini, R.M., Lawrie, C.H., Enblad, G., Larsson, C. *et al.* (2013) Role of microRNAs and microRNA machinery in the pathogenesis of diffuse large B-cell lymphoma. *Blood Cancer J*, **3**, e152.

97. Zheng, M.Z., Sun, X., Li, Y.Q. and Zuo, W.S. (2016) MicroRNA-145 inhibits growth and migration of breast cancer cells through targeting oncoprotein ROCK1. *Tumor Biology*, **37**, 8189-8196.

98. Bradshaw, G., Sutherland, H.G., Haupt, L.M. and Griffiths, L.R. (2016) Dysregulated MicroRNA Expression Profiles and Potential Cellular, Circulating and Polymorphic Biomarkers in Non-Hodgkin Lymphoma. *Genes*, **7**.

99. Mattiske, S., Suetani, R.J., Neilsen, P.M. and Callen, D.F. (2012) The Oncogenic Role of miR-155 in Breast Cancer. *Cancer Epidemiology Biomarkers & Prevention*, **21**, 1236-1243.

100. Li, B., Lu, Y., Wang, H., Han, X., Mao, J., Li, J., Yu, L., Wang, B., Fan, S., Yu, X. *et al.* (2016) miR-221/222 enhance the tumorigenicity of human breast cancer stem cells via modulation of PTEN/Akt pathway. *Biomed Pharmacother*, **79**, 93-101.

101. Liu, X., Lv, X.P., Yang, Q.K., Jin, H.F., Zhou, W.P. and Fan, Q.X. (2018) MicroRNA-29a Functions as a Tumor Suppressor and Increases Cisplatin Sensitivity by Targeting NRAS in Lung Cancer. *Technology in Cancer Research & Treatment*, **17**.

102. Chou, J., Lin, J.H., Brenot, A., Kim, J.W., Provot, S. and Werb, Z. (2013) GATA3 suppresses metastasis and modulates the tumour microenvironment by regulating microRNA-29b expression. *Nat Cell Biol*, **15**, 201-213.

103. Chen, H.X., Xu, X.X., Zhang, Z., Tan, B.Z. and Zhou, X.D. (2017) MicroRNA-29b Inhibits Angiogenesis by Targeting VEGFA through the MAPK/ERK and PI3K/Akt Signaling Pathways in Endometrial Carcinoma. *Cellular Physiology and Biochemistry*, **41**, 933-946.

104. Peng, Y., Guo, J.J., Liu, Y.M. and Wu, X.L. (2014) MicroRNA-34A inhibits the growth, invasion and metastasis of gastric cancer by targeting PDGFR and MET expression. *Bioscience Reports*, **34**, 247-256.

105. Xie, M., Dart, D.A., Guo, T., Xing, X.F., Cheng, X.J., Du, H., Jiang, W.G., Wen, X.Z. and Ji, J.F. (2018) MicroRNA-1 acts as a tumor suppressor microRNA by inhibiting angiogenesis-related growth factors in human gastric cancer. *Gastric Cancer*, **21**, 41-54.

106. Han, C., Zhou, Y.B., An, Q., Li, F., Li, D.L., Zhang, X.J., Yu, Z.J., Zheng, L.L., Duan, Z.F. and Kan, Q.C. (2015) MicroRNA-1 (miR-1) inhibits gastric cancer cell proliferation and migration by targeting MET. *Tumor Biology*, **36**, 6715-6723.

107. Liu, S., Gao, G.Z., Yan, D.X., Chen, X.J., Yao, X.W., Guo, S.Z., Li, G.R. and Zhao, Y. (2017) Effects of miR-145-5p through NRAS on the cell proliferation, apoptosis, migration, and invasion in melanoma by inhibiting MAPK and PI3K/AKT pathways. *Cancer Medicine*, **6**, 819-833.
